# Metabolomics Analysis of the Development of Sepsis and Potential Biomarkers of Sepsis-Induced Acute Kidney Injury

**DOI:** 10.1155/2021/6628847

**Published:** 2021-04-23

**Authors:** Feng Ping, Yingchuan Li, Yongmei Cao, Jiawei Shang, Zhongwei Zhang, Ziming Yuan, Wei Wang, Yong Guo

**Affiliations:** Department of Critical Care Medicine, Shanghai Jiao Tong University Affiliated Sixth People's Hospital, Shanghai 200233, China

## Abstract

Sepsis-induced acute kidney injury (SI-AKI) is a serious condition in critically ill patients. Currently, the diagnosis is based on either elevated serum creatinine levels or oliguria, which partially contribute to delayed recognition of AKI. Metabolomics is a potential approach for identifying small molecule biomarkers of kidney diseases. Here, we studied serum metabolomics alterations in rats with sepsis to identify early biomarkers of sepsis and SI-AKI. A rat model of SI-AKI was established by intraperitoneal injection of lipopolysaccharide (LPS). Thirty Sprague-Dawley (SD) rats were randomly divided into the control (CT) group and groups treated for 2 hours (LPS2) and 6 hours (LPS6) with LPS (10 rats per group). Nontargeted metabolomics screening was performed on the serum samples from the control and SI-AKI groups. Combined multivariate and univariate analysis was used for pairwise comparison of all groups to identify significantly altered serum metabolite levels in early-stage AKI in rats with sepsis. Orthogonal partial least squares discriminant analysis (OPLS-DA) showed obvious separation between the CT and LPS2 groups, CT and LPS6 groups, and LPS2 and LPS6 groups. All comparisons of the groups identified a series of differential metabolites according to the threshold defined for potential biomarkers. Intersections and summaries of these differential metabolites were used for pathway enrichment analysis. The results suggested that sepsis can cause an increase in systemic aerobic and anaerobic metabolism, an impairment of the oxygen supply, and uptake and abnormal fatty acid metabolism. Changes in the levels of malic acid, methionine sulfoxide, and petroselinic acid were consistently measured during the progression of sepsis. The development of sepsis was accompanied by the development of AKI, and these metabolic disorders are directly or indirectly related to the development of SI-AKI.

## 1. Introduction

The clinical mortality rate from sepsis is approximately 20% to 50% and can exceed 70% if sepsis is combined with acute kidney injury [1]. Sepsis-induced acute kidney injury (SI-AKI) is defined as AKI occurring simultaneously with or subsequent to sepsis without other aetiologies [2] and is characterized by high mortality and poor prognosis mainly due to the lack of early and reliable diagnostic markers of AKI, which results in delayed initiation of effective interventions [3]. The serum creatinine (SCR) level and urine volume are affected by many factors, and the sensitivity and specificity of these markers are insufficient. Clinical studies have shown that appropriate measures can be taken to prevent and treat AKI in the early stages of kidney injury [4]. Therefore, identifying new and early markers of kidney injury is required for timely treatment [5]. New biomarkers have been identified in the past few years, including cystatin-C, neutrophil gelatinase-associated lipocalin (NGAL), and interleukin (IL)-18; however, these markers are not sensitive enough to diagnose AKI in the intensive care unit [6].

Metabolomics refers to comprehensive and systematic identification and quantification of all small molecule metabolites in biological samples, such as blood and tissues, under physiological or pathological conditions [7]. Metabolites are the end products of an organism's biochemical activities and provide direct and comprehensive biomarker information that reflects the physiological phenotype. Thus, metabolomics is becoming a powerful analytical approach for studying functional changes in biological systems. Indeed, biomarkers that reflect the changes in cellular metabolism may be identified by metabolomics [8–10].

Our previous study found that during the development of sepsis, the pathological changes corresponding to kidney injury occur earlier than the increases in serum creatinine and urea nitrogen (BUN) levels [11]. Therefore, the present study is aimed at investigating serum metabolomics alterations in rats with SI-AKI and to identify early biomarkers of SI-AKI through gas chromatography/time-of-flight mass spectrometry (GC-TOFMS) for early clinical diagnosis and treatment.

## 2. Materials and Methods

### 2.1. Generation of an Animal Model and Grouping

A rat model of SI-AKI was established based on our previous research [11]. Specific pathogen-free (SPF) male Sprague-Dawley (SD) rats weighing 200-250 g were purchased from the Department of Laboratory Animal Science, Shanghai Jiao Tong University. The present study was approved by the Animal Ethics Committee of Shanghai Jiao Tong University. Thirty SD rats were randomly divided into the control (CT), LPS 2 h (LPS2), and LPS 6 h (LPS6) groups (10 rats per group). Rats in the CT group received an intraperitoneal injection of PBS. In the LPS2 group, the samples were collected 2 hours after intraperitoneal injection of LPS; in the LPS6 group, the samples were collected 6 hours after intraperitoneal injection of LPS. The rats were housed and fed for one week without interventions prior to the treatments. The rats in the LPS2 and LPS6 groups were injected with LPS (dose: 5 mg/kg; concentration: 5 mg/mL; dissolved in PBS), while rats in the CT group were injected with the same volume of PBS solution. The samples were collected from rats anaesthetized with pentobarbital sodium 2 hours and 6 hours after LPS treatment according to the grouping. Blood samples were collected from the inferior vena cava of rats in all groups. The blood was incubated in a centrifuge tube for 30 minutes and then centrifuged for 10 minutes at 3,000 rpm. The serum was collected for metabolomics analysis and biochemical assays. All serum samples were stored in a freezer at -80°C.

### 2.2. Reagents and Instruments

LPS (*Escherichia coli* 055: B5), methoxyamine hydrochloride, fatty acid methyl esters (C7-C30, FAMEs), anhydrous pyridine, and anhydrous sodium sulfate were purchased from Sigma-Aldrich (St. Louis, MO, USA). PBS, the derivatization reagent N-methyl-N-(trimethylsilyl) trifluoroacetamide (MSTFA; containing 1% chlorotrimethylsilane (TMCS)), methanol (Optima; LC-MS grade), and n-ethane were purchased from Thermo Fisher (Fair Lawn, NJ, USA). Dichloromethane, chloroform, and acetone were purchased from the China National Pharmaceutical Group Corporation. An AXSYM automatic immunoassay analyser for liver and kidney function tests was purchased from Abbott Laboratories, Illinois, USA. Ultrapure water was obtained from a Millipore Reference ultrapure water system (Billerica, MA, USA) equipped with a liquid chromatography-coupled 0.22 *μ*m filter. A GC-TOFMS system was purchased from LECO Corp. (St. Joseph, MI, USA).

### 2.3. Histopathological Examination of the Kidney

In the pilot experiments, the kidney tissues were harvested from control rats, rats injected with LPS 2 hours after the injection, and rats injected with LPS 6 hours after the injection, and the samples were used for pathological analysis. Paraffin sections of the kidney tissues were prepared and used for haematoxylin-eosin and periodic acid-Schiff staining.

### 2.4. Biochemical Examination of the Serum

Kidney functional parameters were measured in the serum of rats in all groups by an AXSYM automatic immunoassay analyser.

### 2.5. Metabolomics Analysis

Sample preparation, GC-TOF-MS, and data analysis for serum metabolomics were described in our previous study [12].

#### 2.5.1. Sample Preparation

Serum samples were slowly thawed in a salt-ice bath, and 50 *μ*L of the serum was transferred by a pipettor into a precooled high-speed microcentrifuge tube. Ten microlitres of aqueous solution of an internal standard was added to the mixture, and 200 *μ*L of precooled methanol-chloroform solution was added to extract the supernatant. The supernatant was obtained by centrifugation at 13,500 rpm for 20 minutes at 4°C.

Two hundred microlitres of the serum was transferred to a 1.5 mL autosampling glass vial and placed in a vacuum centrifugal concentrator for 5 minutes. The chloroform was removed, and the content was transferred to a cryogenic freeze-dryer for complete freeze-drying. Next, the samples were returned to room temperature and stored under high-purity nitrogen gas with autocapping. The samples were silylated using the Xplore MET platform. The derivation steps were as follows: addition of 50 *μ*L of methoxyaminopyridine solution, incubation at 30°C for 2 hours, addition of 50 *μ*L of MSTFA, and incubation at 37.5°C for 1 hour. The derivatized samples were automatically injected by an injection arm.

#### 2.5.2. GC-TOFMS Instrument Settings

In GC settings, an Rxi-5MS chromatographic column (30 m, 250 *μ*m × 0.25 *μ*m) was used. The samples (1.0 *μ*L) were injected into the column in splitless mode. Helium was used as a carrier gas at a flow rate of 1.0 mL/minute; the inlet temperature was 270°C, and the transmission interface temperature was 270°C. The programmed temperature conditions of the oven were as follows: 80°C for 2 minutes, an increase to 300°C at 12°C/minute, an increase to 320°C at 4°C/minute over 4.5 minutes, and holding at 320°C for 1 minute.

In MS settings, the ionization mode was electron collision, the electron energy was 70 eV, the detector voltage was 1450 V, and the source temperature was 220°C. The data were acquired in the full-scan mode with the acquisition rate of 25 spectra per second and the mass range of 50-550 Da.

#### 2.5.3. Data Preprocessing

The in-house-developed metabolomics software Xplore MET was used to automatically compare the retention index of deconvoluted peak signals and MS fragment ions with those in JIALIB, the largest database of endogenous metabolites based on silylation derivatization GC-MS (including more than 1500 currently defined endogenous metabolites) [12]. The raw data generated by GC-TOFMS were automatically exported to Xplore MET by ChromaTOF software for baseline smoothing and correction, deconvolution, extraction, and alignment of the original chromatographic peak signals, retention index correction, metabolite identification, and data preprocessing (normalization and standardization).

Xplore MET software was developed by Metabo-Profile Biotechnology, Inc., based on their published pipeline for GC/MS data processing [13] and has been widely used in dozens of published articles [14–18]. The overall data processing procedure is reliable.

### 2.6. Statistical Analysis

SPSS 19.0 software (SPSS, Chicago, IL, USA) was used for statistical analysis, and GraphPad Prism 6.0 (GraphPad Software Inc., San Diego, USA) software was used for plotting. In independent samples *t*-tests, *p* < 0.05 indicated that the difference was statistically significant. Xplore MET software performed data processing, interpretation, and visualization. The data automatically imported into Xplore MET were analysed by multivariate statistical methods, such as principal component analysis (PCA) and orthogonal partial least squares discriminant analysis (OPLS-DA), and by univariate statistical methods, such as Student's *t*-test and the Mann–Whitney *U* test; the model was evaluated according to the relevant parameters.

## 3. Results

### 3.1. Changes in Kidney Function in Rats after Intraperitoneal Injection of LPS

**Figure 1:**
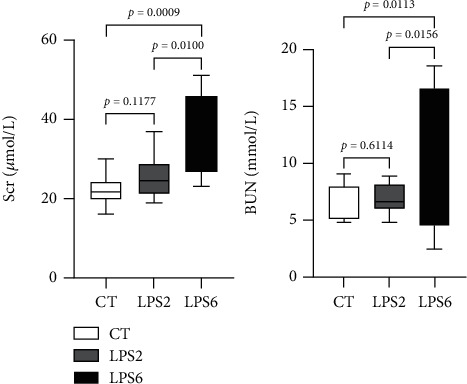
Effect of PBS or LPS treatment on renal function in rats. There were no differences in serum SCR and BUN levels between the LPS2 and control (CT) groups. The serum SCR and BUN levels in the LPS6 group were higher than those in the LPS 2 and control groups (CT).

As shown in Figure 1, comparison with the CT group indicated the lack of significant changes in the serum creatinine and urea nitrogen levels in the LPS2 group (*p* > 0.05); the serum creatinine and urea nitrogen levels were significantly increased in the LPS6 group (*p* = 0.0009 and *p* = 0.0113, respectively); comparison with the LPS2 group indicated that the serum creatinine and urea nitrogen levels were significantly increased in the LPS6 group (*p* = 0.0100 and *p* = 0.0156, respectively).

### 3.2. Pathological Changes in the Kidneys of Rats after Intraperitoneal Injection of LPS

**Figure 2:**
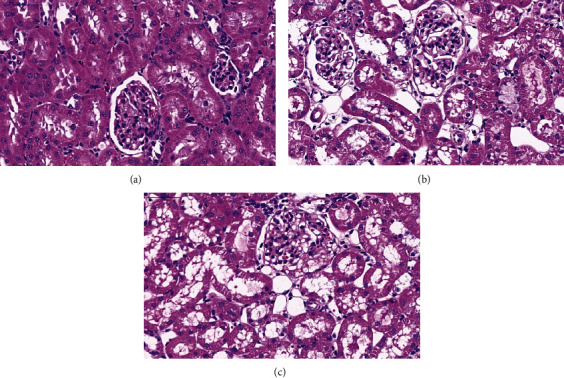
Histological assessment of kidney injury in PBS- or LPS-treated rats by HE staining. (a) HE staining of the kidney tissue in the control (CT) group. (b) HE staining of the kidney tissue in the LPS2 group. (c) HE staining of the kidney tissue in the LPS6 group. HE staining demonstrated that renal tubular epithelial cells in the LPS2 (2B) and LPS6 (2C) groups were denatured and manifested vacuolar degeneration with detachment of the brush border, and the tubular lumen was enlarged compared with those in the control group (2A); necrotic shedding of epithelial cells and tubular formation were detected.

HE staining (Figure 2) demonstrated that renal tubular epithelial cells in the LPS2 (Figure 2(b)) and LPS6 (Figure 2(c)) groups were denatured and manifested vacuolar degeneration with detachment of the brush border, and the tubular lumen was enlarged compared with those in the control group (Figure 2(a)); necrotic shedding of epithelial cells and tubular formation were detected.

**Figure 3:**
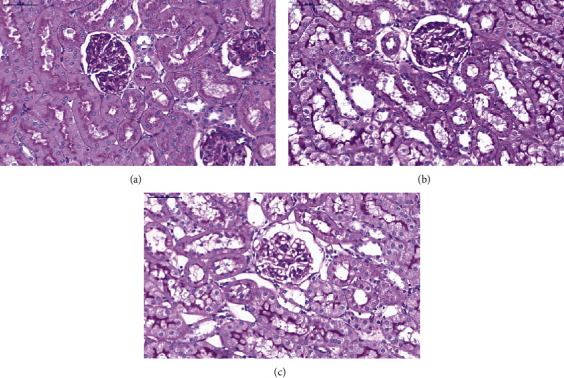
Histological assessment of kidney injury in PBS- or LPS-treated rats by PAS (periodic acid-Schiff) staining. (a) Control (CT) group. (b) LPS2 group. (c) LPS6 group. PAS staining demonstrated that the structure and morphology of the glomerulus, renal tubules, and renal interstitium were normal in the CT group; the staining of the basement membrane indicated full integrity, and abnormal changes, such as inflammatory cell infiltration and fibrosis, were not detected (a). In the LPS2 (b) and LPS6 groups (c), the lumen of the renal tubules was obviously dilated; the staining of the basement membrane of tubular epithelial cells was discontinuous, and epithelial cells were irregular and of different sizes. The arrangement of renal tubular epithelial cells was more disordered than that in the CT group, and cell shedding was detected.

PAS staining (Figure 3) demonstrated that the structure and morphology of the glomerulus, renal tubules, and renal interstitium were normal in the CT group; the staining of the basement membrane indicated full integrity, and abnormal changes, such as inflammatory cell infiltration and fibrosis, were not detected (Figure 3(a)). In the LPS2 (Figure 3(b)) and LPS6 groups (Figure 3(c)), the lumen of the renal tubules was obviously dilated; the staining of the basement membrane of tubular epithelial cells was discontinuous, and epithelial cells were irregular and of different sizes. The arrangement of renal tubular epithelial cells was more disordered than that in the CT group, and cell shedding was detected.

### 3.3. Pairwise Comparisons (CT vs. LPS2)

An overview of metabolite classifications is shown in Supplementary Figure 1. Multivariate statistical analysis (CT vs. LPS2) according to the principal component analysis (PCA) and orthogonal partial least squares discriminant analysis (OPLS-DA) models is shown in Supplementary Figures 2 and 3. Supplementary Figures 4 and 5 summarize differential metabolites identified by univariate statistical analysis.

**Figure 4:**
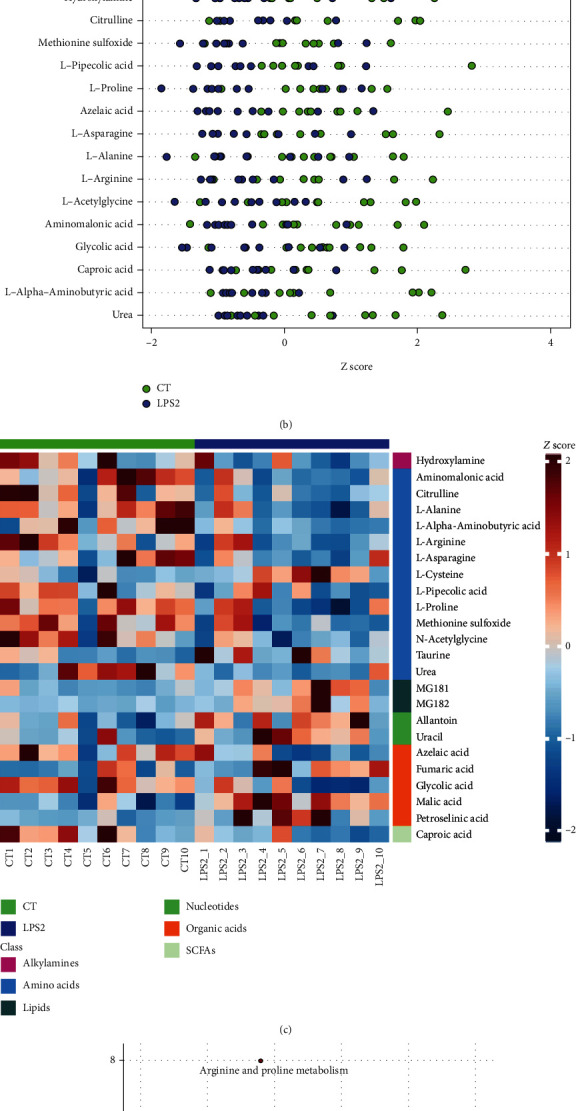
(a) Venn diagram of differential metabolites identified by multivariate and univariate statistical methods. (b) *Z* score plots of 24 potential biomarkers. (c) *Z* score heat map of 24 potential biomarkers according to the classes with names (CT vs. LPS 2). (d) Overview of pathway analysis. Metabolic pathway enrichment analysis (MPEA) was performed to identify the most relevant metabolic pathways.

The Venn diagram of differential metabolites identified by multivariate and univariate statistical analyses is shown in Figure 4(a). In this analysis, the screening criteria for potential biomarkers were as follows: (1) in univariate statistical analysis, *p* < 0.05 and ∣log2fc | >0, and (2) in multivariate statistical analysis, VIP > 1. The *Z* score plots of 24 potential biomarkers are shown in Figure 4(b).  Figure 4(c) shows the *Z* score heat map of these potential biomarkers labelled according to the types of metabolites. In pathway analysis (CT vs. LPS2), the differential metabolites included potential biomarkers defined by the screening criteria. The results of pathway enrichment analysis for these differential metabolites are summarized in Figure 4(d) and Table 1.

### 3.4. Pairwise Comparisons (LPS2 vs. LPS6)

An overview of metabolite classifications is shown in Supplementary Figure 6. Multivariate statistical analysis (LPS2 vs. LPS6) according to PCA and OPLS-DA models is shown in Supplementary Figures 7 and 8. Supplementary Figures 9 and 10 summarize differential metabolites obtained by univariate statistical analysis.

**Figure 5:**
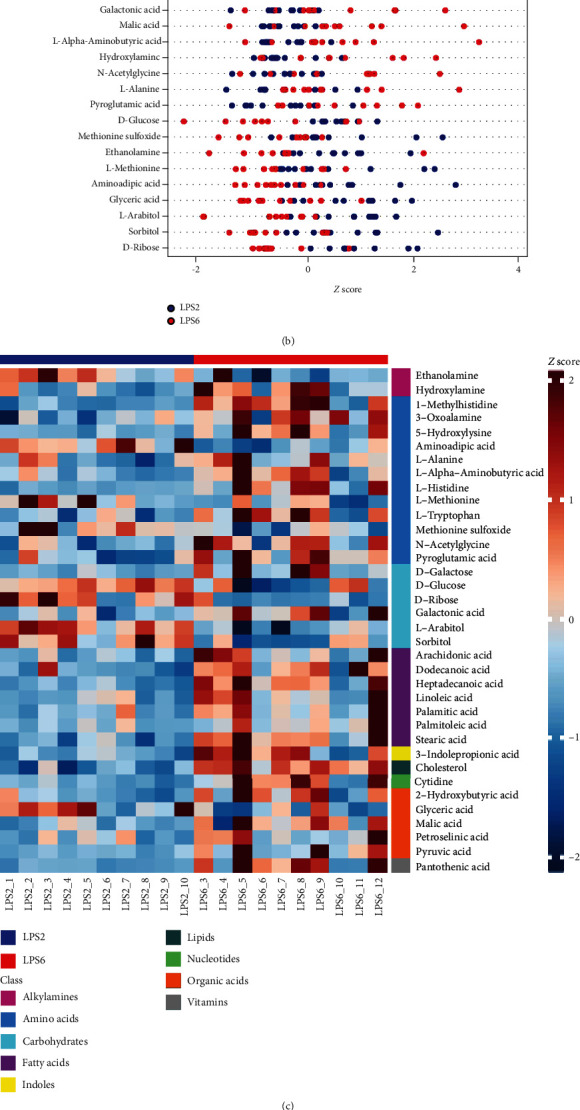
(a) Venn diagram of differential metabolites identified by multivariate and univariate statistical methods. (b) Z-sZ score plots of 36 potential biomarkers. (c) Z-sZ score heat map of 36 potential biomarkers according to the classes with names (LPS2 vs. LPS6). (d) Overview of pathway analysis. Metabolic pathway enrichment analysis (MPEA) was performed to identify the most relevant metabolic pathways.

The Venn diagram of differential metabolites identified by multivariate and univariate statistical analyses is shown in Figure 5(a). The screening criteria for potential biomarkers were as follows: (1) in univariate statistical analysis, *p* < 0.05 and ∣log2fc | >0, and (2) in multivariate statistical analysis, VIP > 1. The *Z* score plots of 36 potential biomarkers are shown in Figure 5(b).  Figure 5(c) shows the *Z* score heat map of these potential biomarkers labelled by the types of metabolites. In pathway analysis (LPS2 vs. LPS6), the differential metabolites included potential biomarkers defined by the screening criteria. The results of pathway enrichment analysis for these differential metabolites are summarized in Figure 5(d) and Table 2.

### 3.5. Pairwise Comparisons (CT vs. LPS6)

An overview of metabolite classifications is shown in Supplementary Figure 11. Multivariate statistical analysis (CT vs. LPS6) according to PCA and OPLS-DA models is shown in Supplementary Figures 12 and 13. Supplementary Figures 14 and 15 summarize differential metabolites identified by univariate statistical analysis.

**Figure 6:**
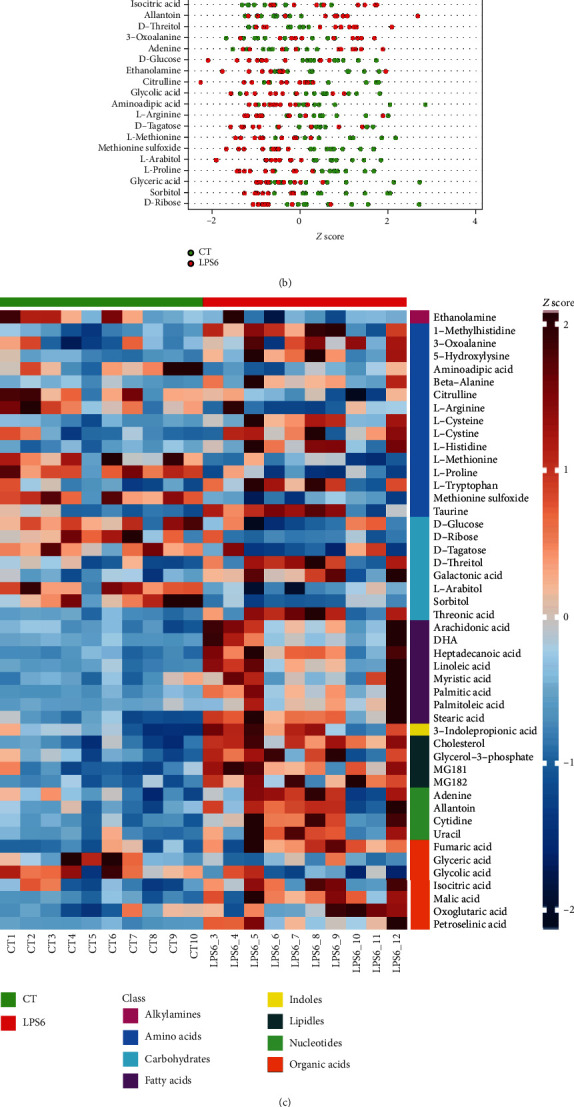
(a) Venn diagram of differential metabolites identified by multivariate and univariate statistical methods. (b) *Z* score plots of 48 potential biomarkers. (c) *Z* score heat map according to the classes with names (CT vs. LPS6). (d) Overview of pathway analysis. Metabolic pathway enrichment analysis (MPEA) was performed to identify the most relevant metabolic pathways.

In screening of potential biomarkers (CT vs. LPS6), the Venn diagram of differential metabolites identified by multivariate and univariate statistical analyses is shown in Figure 6(a). The screening criteria for potential biomarkers were as follows: (1) in univariate statistical analysis, *p* < 0.05 and ∣log2fc | >0, and (2) in multivariate statistical analysis, VIP > 1. The *Z* score plots of 48 potential biomarkers are shown in Figure 6(b).  Figure 6(c) shows the *Z* score heat map of these 48 potential biomarkers labelled according to the types of metabolites. In pathway analysis (control vs. LPS6), the differential metabolites included potential biomarkers defined by the screening criteria. The results of pathway enrichment analysis for these differential metabolites are summarized in Figure 6(d) and Table 3.

### 3.6. Comparison of All Three Groups (CT vs. LPS2 vs. LPS6)

An overview of metabolite classifications is shown in Supplementary Figure 16. Multivariate statistical analysis (CT vs. LPS2 vs. LPS6) according to PCA and PLS-DA models is shown in Supplementary Figures 17 and 18. Supplementary Figures 19 and 20 summarize differential metabolites obtained by univariate statistical analysis.

**Figure 7:**
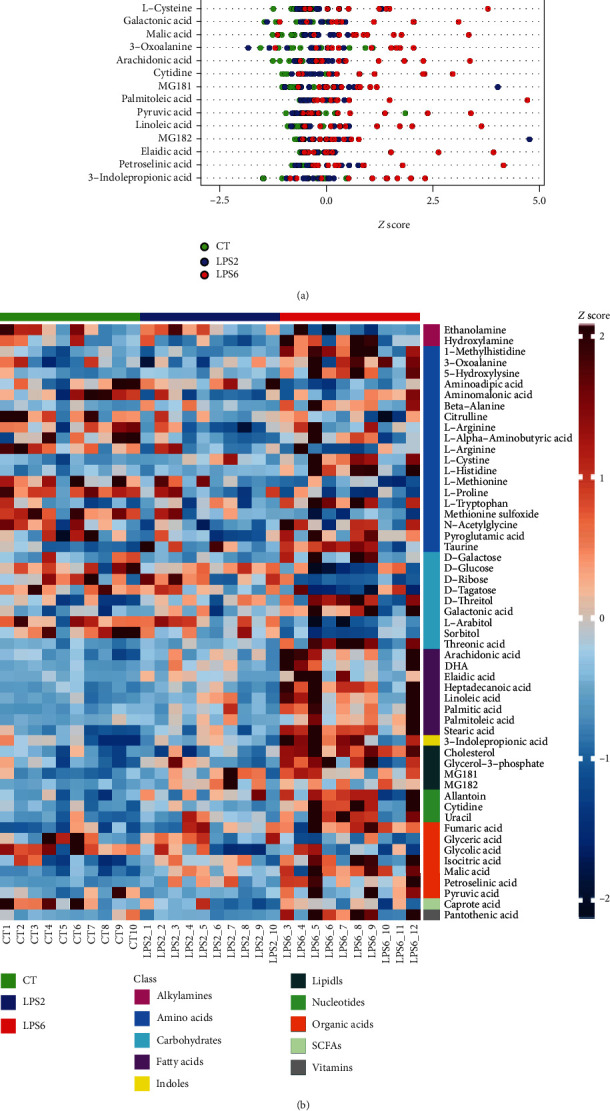
(a) *Z* score plots of 55 potential biomarkers. (b) *Z* score heat map of 55 potential biomarkers according to the classes with names (CT vs. LPS2 vs. LPS6). (c) Overview of pathway analysis. Metabolic pathway enrichment analysis (MPEA) was performed to identify the most relevant metabolic pathways.

In screening of potential biomarkers (CT vs. LPS2 vs. LPS6), this comparison was performed in multiple groups; thus, OPLS-DA cannot be used. Hence, potential biomarkers included differential metabolites identified by univariate statistical analysis. The *Z* score plots of 55 potential biomarkers are shown in Figure 7(a).  Figure 7(b) shows the *Z* score heat map of these 55 potential biomarkers labelled according to the types of metabolites. In pathway analysis (CT vs. LPS2 vs. LPS6), the differential metabolites included potential biomarkers defined by the screening criteria. The results of pathway enrichment analysis for these differential metabolites are summarized in Figure 7(c) and Table 4.

### 3.7. Summary of Differential Metabolites

**Figure 8:**
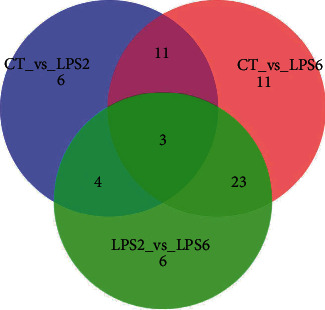
Summary of differential metabolites. The circles of different colour represent different comparisons. The overlapping parts of the circles represent the intersections of the corresponding comparisons. The number in each region indicates the number of differential metabolites in the corresponding set. The data indicated that three metabolites were consistently significantly different during the development of SI-AKI. These three metabolites, malic acid, methionine sulfoxide and petroselinic acid, are potential biomarkers for SI-AKI.

Each group comparison identified a series of differential metabolites (potential biomarkers) according to defined thresholds for potential biomarkers. The results of each group comparison identifying differential metabolites were summarized, and the summary Venn diagram (or petal diagram) of the differential metabolites is shown in Figure 8. The data indicated that three metabolites were consistently significantly different during the development of SI-AKI. These three metabolites, malic acid, methionine sulfoxide, and petroselinic acid, are potential biomarkers for SI-AKI.

## 4. Discussion

In this study, our data showed that in rats, pathological changes occurred in the kidneys 2 hours after intraperitoneal injection of LPS, but renal function indicators, such as serum creatinine and urea nitrogen levels, were not changed until 6 hours after LPS injection. Additionally, we used a metabolomics platform to identify three metabolites that were consistently changed during the development of SI-AKI: malic acid, methionine sulfoxide, and petroselinic acid. Accordingly, the dysregulated metabolic pathways associated with SI-AKI included the citric acid cycle, oxidative stress pathways, and fatty acid metabolism pathways. These altered metabolic pathways are directly or indirectly associated with SI-AKI.

Malic acid is an important organic acid produced during metabolic processes in the body and an important metabolic intermediate in the tricarboxylic acid (TCA) cycle that directly participates in mitochondrial energy metabolism [19]. Malic acid is an important component of the malate-aspartate shuttle and plays an important role in regulating the transfer of reducing equivalents between the cytosol and mitochondria [20]. As the malic acid concentration increases, mitochondrial ATP synthesis is enhanced, and the utilization of ATP is also enhanced [21]. An increase in the malic acid concentration indicates an increase in the concentration of the TCA substrates and in flux through the TCA cycle. In addition, an increase in the malic acid concentration enhances the effects on the malate-aspartate shuttle and the amount of NADH in the mitochondrial matrix. An increase in the mitochondrial respiratory rate may be related to the combined effects of these two events [20]. Animal experiments have shown that when the body's ATP demand increases, the concentration of malic acid in the liver mitochondria increases, and the role of mitochondria in ATP synthesis from the corresponding substrates is intensified. Malic acid may be the driving force for ATP production in mitochondria [21]. A stress response can occur in rats with sepsis. This stress-induced metabolic response involves the neuroendocrine, inflammatory, and immune systems. Stress-induced oxygen metabolism is characterised by increased oxygen consumption and decreased efficiency of the oxygen supply. Our results showed that with the development of SI-AKI, the intermediate products of the TCA cycle, such as malic acid, fumaric acid, isocitric acid, and pyruvic acid, increased. In addition, we observed a gradual increase in the lactic acid levels. These data suggested that rats with SI-AKI manifested increased oxygen requirements and an increase in both aerobic and anaerobic respiration. Moreover, these data indicated that as SI-AKI progresses, the imbalance in oxygen metabolism becomes more severe.

Methionine is one of the most vulnerable amino acids in proteins, and its oxidation causes a series of changes in the protein structure, function, and signal transduction, which are related to many diseases and conditions, such as ageing, cancer, and neurodegenerative diseases [22]. Methionine sulfoxide is an oxidation product of a reaction of methionine with reactive oxygen species via a 2-electron mechanism. Such oxidants can be generated by activated neutrophils; therefore, methionine sulfoxide can be regarded as a biomarker of oxidative stress in vivo [23]. Numerous studies have shown pathological dependence on oxygen supply in experimental animals during the development of sepsis. This pathological dependence occurs because oxygen uptake cannot meet the oxygen demand of the tissue and manifests as dysregulation of systemic tissue uptake and oxygen utilization [24, 25]. In the early stage of sepsis, blood flow is abnormally distributed throughout the body, which may lead to a relative excess of blood flow in some organs with low metabolism, while organs with high metabolism have insufficient blood flow, resulting in tissue hypoxia and a decrease in VO_2_. Moreover, endotoxin and some inflammatory mediators impair the regulation of microcirculation, leading to the formation of microthromboses and a decrease in the capillary density. These processes result in insufficient DO_2_ and increased capillary permeability manifested as interstitial leakage and oedema and consequent oxygen diffusion from microvessels to the cells. Subsequent damage to cytoskeletal filaments aggravates the dysfunction of oxygen uptake and utilization [26, 27]. Andersen et al. demonstrated that the level of methionine sulfoxide is decreased during the development of sepsis in patients with septic shock [28]. In septic rats in the present study, serum methionine sulfoxide levels were gradually decreased during the development of septic AKI. These findings suggested that a wide range of oxygen supply and oxygen uptake disorders affect tissues and organs throughout the body.

Lucchi et al. demonstrated that patients with uraemia often have abnormal lipid metabolism [29]. Deng et al. reported that 14(15)-epoxyeicosatrienoic acid (14(15)-EET) and 19(20)-epoxydocosapentaenoic acid (19(20)-EDP), the major epoxide metabolites of arachidonic acid (ARA) and docosahexaenoic acid (DHA), respectively, had contrasting effects on kidney injury in a mouse model of ischaemia/reperfusion- (I/R-) induced AKI. Specifically, 14(15)-EET mitigated and 19(20)-EDP exacerbated I/R kidney injury [30]. Moran et al. demonstrated that cytochrome P450-mediated epoxidation of linoleic acid in a rabbit renal proximal tubule model produced biologically active metabolites that resulted in acute renal failure [31]. Concentration-dependent studies have shown that linoleic acid and linoleic acid monoepoxides are the most toxic species that induce mitochondrial dysfunction prior to cell death [31]. Lucchi et al. demonstrated that the levels of retinol are increased in patients with end-stage chronic renal failure (ESCRF) due to reduced excretion of retinol-binding protein and detected a significant increase in the levels of conjugated linoleic acid (CLA), palmitoleic acid (16:1) and oleic acid (18:1) in the plasma samples from these patients because retinol influences lipid metabolism [32]. Istvan et al. demonstrated that oleic acid increased ROS production in renal proximal tubule cells via mitochondria and, to a lesser extent, via NADPH oxidase, resulting in ROS-dependent mitochondrial depolarization and consequent injury [33]. Petroselinic acid is a fatty acid that can enhance mitochondrial activity in humans [34]. Petroselinic acid is involved in arachidonic acid metabolism, and an increase in its concentration may be related to immune inflammation [35]. The data of the present study indicated that the levels of docosahexaenoic acid, arachidonic acid, linoleic acid, oleic acid, palmitic acid, and stearic acid were gradually increased during the development of SI-AKI and that the serum concentration of petroselinic acid was increased during the onset of SI-AKI. Abnormalities in lipid metabolism, especially elevated serum concentration of petroselinic acid, may be early serum markers of SI-AKI, which are more sensitive than traditional renal functional indicators, such as serum creatinine level.

## 5. Conclusion

In conclusion, we used metabolomics techniques to demonstrate that sepsis could cause an increase in systemic aerobic and anaerobic metabolism, impairments in oxygen supply, and uptake and abnormal fatty acid metabolism. In particular, changes in malic acid, methionine sulfoxide, and petroselinic acid consistently occur during the progression of sepsis. The development of sepsis was accompanied by the development of AKI, and these metabolic disorders are directly or indirectly related to the development of SI-AKI. Our understandings of the metabolic disorders described herein and their underlying mechanisms are far from clear. The exact mechanism between these metabolic alterations and sepsis development need to be validated subsequently by animal experiments with a larger sample.

## Figures and Tables

**Table 1:** Differential metabolic pathways (CT vs. LPS2). The results of enriched compounds after screening and metabolic metabolic pathway enrichment analysis (MPEA) between CT and LPS2 groups are summarized in this table.

	Total in pathway	Expected	Hits	Raw P	′ − log(*p*)	Holm P	FDR	Impact	Enriched compounds
Arginine and proline metabolism	44	0.65906	5	0.000338	7.9912	0.027413	0.027413	0.18368	Citrulline Fumaric acid L-Arginine L-Proline Urea
Aminoacyl-tRNA biosynthesis	67	1.0036	5	0.002405	6.0301	0.19243	0.097416	0.10415	L-Alanine L-Arginine L-Asparagine L-Cysteine L-Proline
Alanine, aspartate, and glutamate metabolism	24	0.35949	3	0.004792	5.3407	0.3786	0.11482	0.04	Fumaric acid L-Alanine L-Asparagine
Taurine and hypotaurine metabolism	8	0.11983	2	0.00567	5.1726	0.44225	0.11482	0.5	L-Cysteine Taurine
Pantothenate and CoA biosynthesis	15	0.22468	2	0.019959	3.9141	1	0.32333	0.07692	L-Cysteine Uracil

Holm: Holm-Bonferroni method; FDR: false discovery rate.

**Table 2:** Differential metabolic pathways (LPS2 vs. LPS6). The results of enriched compounds after screening and metabolic pathway enrichment analysis (MPEA) between the LPS2 and LPS6 groups are summarized in this table.

	Total in pathway	Expected	Hits	Raw P	′ − log(*p*)	Holm P	FDR	Impact	Enriched compounds
Biosynthesis of unsaturated fatty acids	42	0.95863	4	0.013612	4.2968	1	1	0	Arachidonic acid Linoleic acid Palmitic acid Stearic acid
Histidine metabolism	15	0.34237	2	0.044048	3.1225	1	1	0.23077	1-Methylhistidine L-Histidine

Holm: Holm-Bonferroni method; FDR: false discovery rate.

**Table 3:** Differential metabolic pathways (CT vs. LPS6). The results of enriched compounds after screening and metabolic pathway enrichment analysis (MPEA) between the CT and LPS6 groups are summarized in this table.

	Total in pathway	Expected	Hits	Raw P	′ − log(*p*)	Holm P	FDR	Impact	Enriched compounds
Biosynthesis of unsaturated fatty acids	42	1.2882	5	0.007835	4.8492	0.63462	0.28969	0	(4Z,7Z,10Z,13Z,16Z,19Z)-Docosahexaenoic acid Arachidonic acid Linoleic acid Palmitic acid Stearic acid
Pantothenate and CoA biosynthesis	15	0.46006	3	0.009458	4.6609	0.75666	0.28969	0.07692	Beta-alanine L-Cysteine Uracil
Glyoxylate and dicarboxylate metabolism	16	0.49073	3	0.011394	4.4747	0.90014	0.28969	0.16666	Glyceric acid Glycolic acid Isocitric acid
Aminoacyl-tRNA biosynthesis	67	2.0549	6	0.014306	4.2471	1	0.28969	0.12498	L-Arginine L-Cysteine L-Histidine L-Methionine L-Proline L-Tryptophan
Citrate cycle (TCA cycle)	20	0.61341	3	0.021295	3.8493	1	0.30896	0.10345	Fumaric acid Isocitric acid Oxoglutaric acid
Taurine and hypotaurine metabolism	8	0.24536	2	0.022886	3.7772	1	0.30896	0.5	L-Cysteine Taurine
Arginine and proline metabolism	44	1.3495	4	0.042713	3.1532	1	0.49425	0.18368	Citrulline Fumaric acid L-Arginine L-Proline

Holm: Holm-Bonferroni method; FDR: false discovery rate.

**Table 4:** Differential metabolic pathways (CT vs. LPS2 vs. LPS6). The results of enriched compounds after screening and metabolic pathway enrichment analysis (MPEA) (CT vs. LPS2 vs. LPS6) are summarized in this table.

	Total in pathway	Expected	Hits	Raw P	′ − log(*p*)	Holm P	FDR	Impact	Enriched compounds
Pantothenate and CoA biosynthesis	15	0.52425	4	0.001355	6.6038	0.10977	0.10864	0.15384	Beta-alanine L-Cysteine Pantothenic acid Uracil
Biosynthesis of unsaturated fatty acids	42	1.4679	6	0.002682	5.9211	0.21459	0.10864	0	(4Z,7Z,10Z,13Z,16Z,19Z)-Docosahexaenoic acid Arachidonic acid Linoleic acid Oleic acid Palmitic acid Stearic acid
Aminoacyl-tRNA biosynthesis	67	2.3417	7	0.007184	4.936	0.5675	0.19395	0.14581	L-Alanine L-Arginine L-Cysteine L-Histidine L-Methionine L-Proline L-Tryptophan
Glyoxylate and dicarboxylate metabolism	16	0.5592	3	0.016309	4.116	1	0.33026	0.16666	Glyceric acid Glycolic acid Isocitric acid
Taurine and hypotaurine metabolism	8	0.2796	2	0.029294	3.5304	1	0.40633	0.5	L-Cysteine Taurine
Citrate cycle (TCA cycle)	20	0.699	3	0.030099	3.5033	1	0.40633	0.13794	Fumaric acid Isocitric acid Pyruvic acid
Alanine, aspartate, and glutamate metabolism	24	0.8388	3	0.048471	3.0268	1	0.56088	0.04	Fumaric acid L-Alanine Pyruvic acid

Holm: Holm-Bonferroni method; FDR: false discovery rate.

## Data Availability

All the data used to support the findings of this study are included within the article. Please address all requests about the data to the authors.

## Supplementary Materials

Supplementary Materials Supplementary Figure 1. Classification of metabolites in the control (CT) and LPS 2 h (LPS2) groups. The stacked histogram showing the relative abundance statistics of the median values of various metabolites in each group of samples is shown in Supplementary Figure 1(A). The stacked histogram showing the relative abundances of various types of metabolite in each sample is shown in Supplementary Figure 1(B). Supplementary Figure 2: PCA score plots for the CT and LPS2 groups. The 2D and 3D PCA scores are shown in Supplementary Figures 2A and 2B, respectively. Supplementary Figure 2(C) shows the 2D PCA score plot for analysed samples and box plots corresponding to the principal component scores. Supplementary Figure 3: OPLS-DA score plot, permutation test results, and volcano plot: the OPLS-DA 2D score plot is shown in Supplementary Figure 3(A); the permutation test results are shown in Supplementary Figure 3(B). Visualization of differential metabolite profiles by volcano plot is shown in Supplementary Figure 3(C). Supplementary Figure 4(A): enhanced volcano plot and *Z*-score plot showing differential metabolites selected by univariate analysis. The volcano plot shows differential metabolites identified by univariate statistical analysis (Supplementary Figure 4(A)). The threshold settings for the volcano plot were as follows: (1) *p* < 0.05 and (2) an absolute value of log2fc > 0 (where fc is the fold change, i.e., the factor of the intergroup change). Highlighted metabolites in the upper right corner were increased, and highlighted metabolites in the upper left corner were decreased in the LPS2 group compared with those in the CT group (Supplementary Figure 4(A)). The screening criteria were used to identify 25 differential metabolites by univariate statistical analysis (*p* < 0.05). Supplementary Figure 4(B) shows the *Z*-score plot of these 25 differential metabolites. Supplementary Figure 5: top ranking differential metabolites between the two groups. Nine representative differential metabolites (top ranking) identified by univariate statistical analysis and the corresponding *p* value rankings are shown. Supplementary Figure 6: classification of metabolites in the LPS 2 h (LPS2) and LPS 6 h (LPS6) groups. The stacked histogram showing the relative abundance statistics of the median values of various metabolites in each group of samples (Supplementary Figure 6(A)). The stacked histogram showing the relative abundances of various types of metabolites in each sample (Supplementary Figure 6(B)). Supplementary Figure 7: PCA score plots for the LPS2 and LPS6 groups. The 2D and 3D PCA scores are shown in Supplementary Figures 7(A) and 7(B), respectively. Supplementary Figure 7(C) shows the 2D PCA score plot of the analysed samples and box plots corresponding to the principal component scores. Supplementary Figure 8: OPLS-DA score plot, permutation test results, and volcano plot. The OPLS-DA 2D score plot is shown in Supplementary Figure 8(A); the permutation test results are shown in Supplementary Figure 8(B); visualization of differential metabolite profiles by volcano plot is shown in Supplementary Figure 8(C). Supplementary Figure 9: enhanced volcano plot and *Z* Z sscore plot showing differential metabolites identified by multivariate analysis. The volcano plot shows differential metabolites identified by univariate statistical analysis (Supplementary Figure 9(A)). In this analysis, the threshold settings for the volcano plot were as follows: (1) *p* < 0.05 and (2) an absolute value of log2fc > 0 (where fc is the fold change, i.e., the factor of the intergroup change). Highlighted metabolites in the upper right corner were increased, and highlighted metabolites in the upper left corner were decreased in the LPS6 group compared with those in the LPS2 group (Supplementary Figure 9(A)). The screening criteria were used to identify 38 differential metabolites by univariate statistical analysis (*p* < 0.05). Supplementary Figure 9(B) shows the *Z* score plot of these 38 differential metabolites. Supplementary Figure 10: top ranking differential metabolites between the two groups. Nine representative differential metabolites (top ranking) identified by univariate statistical analysis and their *p* value rankings are shown. Supplementary Figure 11: classification of metabolites in the CT and LPS 6 h (LPS6) groups. The stacked histogram showing the relative abundance statistics of the median values of various metabolites in each group of samples (Supplementary Figure 11(A)). The stacked histogram showing the relative abundances of various types of metabolites in each sample (Supplementary Figure 11(B)). Supplementary Figure 12: PCA score plots for the CT and LPS6 groups. The 2D and 3D PCA scores are shown in Supplementary Figures 12(A) and 12(B), respectively. Supplementary Figure 12(C) shows the 2D PCA score plot of the analysed samples and box plots corresponding to the principal component scores. Supplementary Figure 13: OPLS-DA score plot, permutation test results, and volcano plot. Individual metabolic profiles from the two predefined groups were differentiated using a more sophisticated multivariate statistical OPLS-DA model (Supplementary Figure 13(A)). Permutation testing (see Materials and Methods for details) was used to validate the classification model (Supplementary Figure 13(B)). A volcano plot (Supplementary Figure 13(C)) was used to identify reliable metabolic markers. Supplementary Figure 14(A): enhanced volcano plot showing differential metabolites identified by univariate analysis; (B) *Z* score plot of these 51 differential metabolites. The volcano plot shows differential metabolites identified by univariate statistical analysis (Supplementary Figure 14(A)). The threshold settings for the volcano plot were as follows: (1) *p* < 0.05 and (2) an absolute value of log2fc > 0 (where fc is the fold change, i.e., the factor of the intergroup change). Highlighted metabolites in the upper right corner were increased, and highlighted metabolites in the upper left corner were decreased in the LPS6 group compared with those in the CT group (Supplementary Figure 14(A)). According to the screening criteria, 51 differential metabolites were identified by univariate statistical analysis. Supplementary Figure 14(B) shows the *Z* score plot of these 51 differential metabolites. Supplementary Figure 15: top ranking differential metabolites between the two groups. Nine representative differential metabolites (top ranking) identified by univariate statistical analysis and the corresponding *p* value rankings are shown. Supplementary Figure 16: classification of metabolites in the CT, LPS2, and LPS6 groups. The stacked histogram showing the relative abundance statistics of the median values of various metabolites in each group of samples (Supplementary Figure 16(A)). The stacked histogram showing the relative abundances of various types of metabolites in each sample (Supplementary Figure 16(B)). Supplementary Figure 17: PCA score plots for the CT, LPS2, and LPS6 groups. The 2D and 3D PCA scores are shown in Supplementary Figures 17(A) and 17(B), respectively. Supplementary Figure 17(C) shows the 2D PCA score plot of the analysed samples and box plots corresponding to the principal component scores. Supplementary Figure 18: PLS-DA score plot of subject classifications. (A) 2D PLS-DA score plot, (B) 3D PLS-DA score plot, (C) PLS-DA score plot with corresponding box plots. Widely used supervised multivariate classification model, PLS-DA, was used to compare multiple groups because this model maximizes the intergroup differences (Supplementary Figure 18(A), 2D PLS-DA score plot; Supplementary Figure 18(B), 3D PLS-DA score plot). Supplementary Figure 18(C) shows the PLS-DA score plot with the corresponding box plots. Supplementary Figure 19: *Z* score plot of 55 differential metabolites. These 55 differential metabolites were identified by univariate statistical analysis. Supplementary Figure 19 shows the *Z* score plot of these 55 differential metabolites. Supplementary Figure 20: top ranking differential metabolites among the three groups. Nine representative differential metabolites (top ranking) identified by univariate statistical analysis and their *p* value rankings are shown.
