# Supplementary material for: Metabolomics Analysis of the Development of Sepsis and Potential Biomarkers of Sepsis-Induced Acute Kidney Injury
Source: Oxid Med Cell Longev. 2021 Apr 23;2021:6628847. doi: 10.1155/2021/6628847 (PMC8088350; doi:10.1155/2021/6628847)
Supplement: Supplementary Materials — Supplementary Figure 1. Classification of metabolites in the control (CT) and LPS 2 h (LPS2) groups. The stacked histogram showing the relative abundance statistics of the median values of various metabolites in each group of samples is shown in Supplementary Figure 1(A). The stacked histogram showing the relative abundances of various types of metabolite in each sample is shown in Supplementary Figure 1(B). Supplementary Figure 2: PCA score plots for the CT and LPS2 groups. The 2D and 3D PCA scores are shown in Supplementary Figures 2A and 2B, respectively. Supplementary Figure 2(C) shows the 2D PCA score plot for analysed samples and box plots corresponding to the principal component scores. Supplementary Figure 3: OPLS-DA score plot, permutation test results, and volcano plot: the OPLS-DA 2D score plot is shown in Supplementary Figure 3(A); the permutation test results are shown in Supplementary Figure 3(B). Visualization of differential metabolite profiles by volcano plot is shown in Supplementary Figure 3(C). Supplementary Figure 4(A): enhanced volcano plot and Z-score plot showing differential metabolites selected by univariate analysis. The volcano plot shows differential metabolites identified by univariate statistical analysis (Supplementary Figure 4(A)). The threshold settings for the volcano plot were as follows: (1) p < 0.05 and (2) an absolute value of log2fc > 0 (where fc is the fold change, i.e., the factor of the intergroup change). Highlighted metabolites in the upper right corner were increased, and highlighted metabolites in the upper left corner were decreased in the LPS2 group compared with those in the CT group (Supplementary Figure 4(A)). The screening criteria were used to identify 25 differential metabolites by univariate statistical analysis (p < 0.05). Supplementary Figure 4(B) shows the Z-score plot of these 25 differential metabolites. Supplementary Figure 5: top ranking differential metabolites between the two groups. Nine representativ [file 6628847.f1.docx]

**Metabolomics analysis of sepsis development and potential biomarkers of sepsis-induced acute kidney injury**

**Feng Ping,^1^ Yong Guo,^1^ Yongmei Cao,^1^ Jiawei Shang,^1^ Zhongwei Zhang,^1^ Ziming Yuan,^1^ Wei Wang,^1^ Yingchuan Li^1^**

*^1^Department of Critical Care Medicine, Shanghai Jiao Tong University Affiliated Sixth People’s Hospital, Shanghai 200233, China.*

Feng Ping and Yong Guo are equal contributor.

Correspondence should be addressed to Yingchuan Li

E-mail [Yingchuan.li@sjtu.edu.cn](mailto:Yingchuan.li@sjtu.edu.cn)

Yong Guo

E-mail [Garfield.guo@sjtu.edu.cn](mailto:Garfield.guo@sjtu.edu.cn)

**SUPPLEMENTARY FIGURES**

**
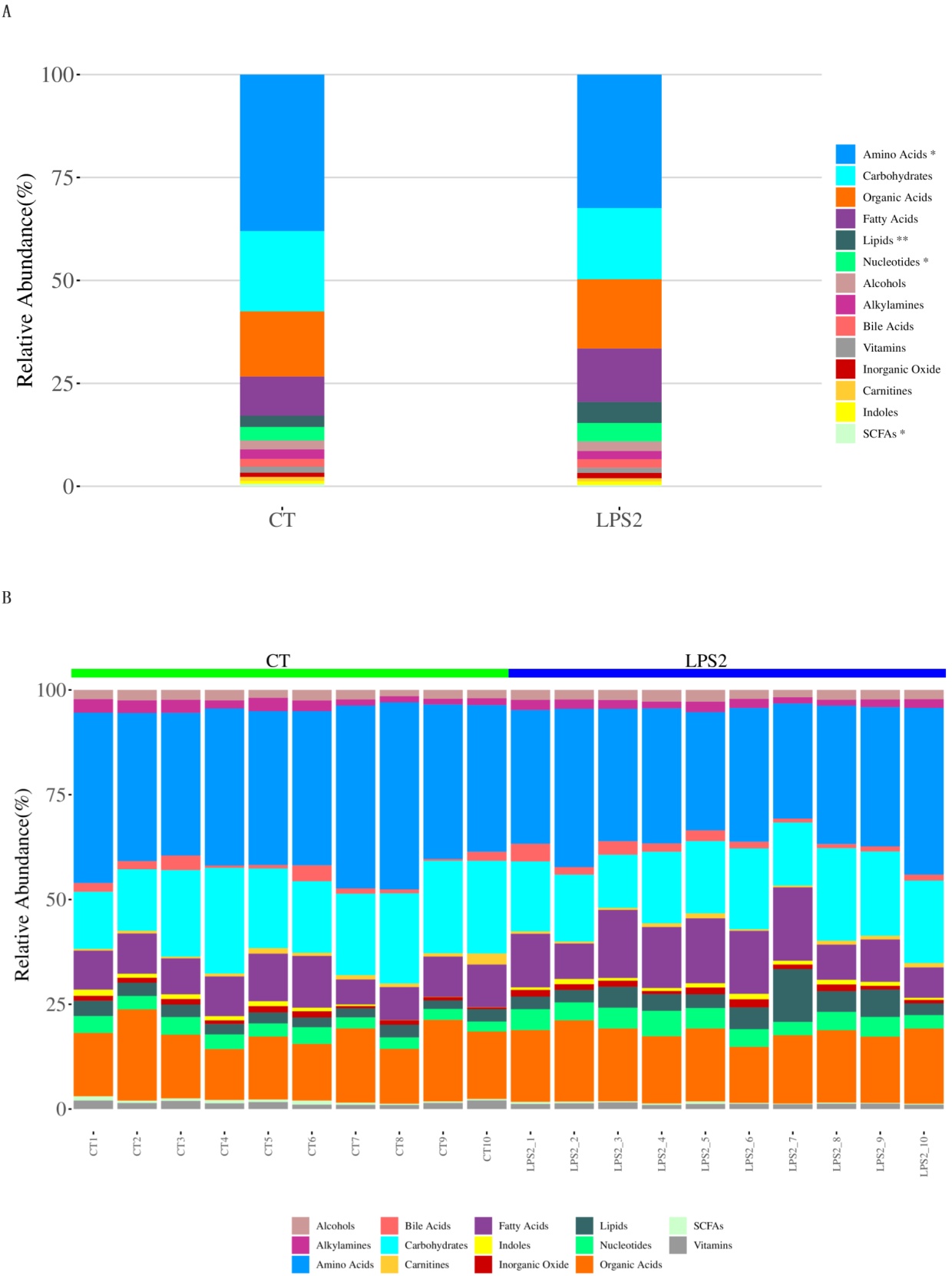
**

**Supplementary Figure 1 Classification of metabolites in the CT group and LPS2 group.** The stacked histogram showing the relative abundance statistics of the median value of various metabolites in each group of samples is shown in **Supplementary Figure 1A**. The stacked histogram showing the relative abundances of various metabolite types in each sample is shown in **Supplementary Figure 1B**.


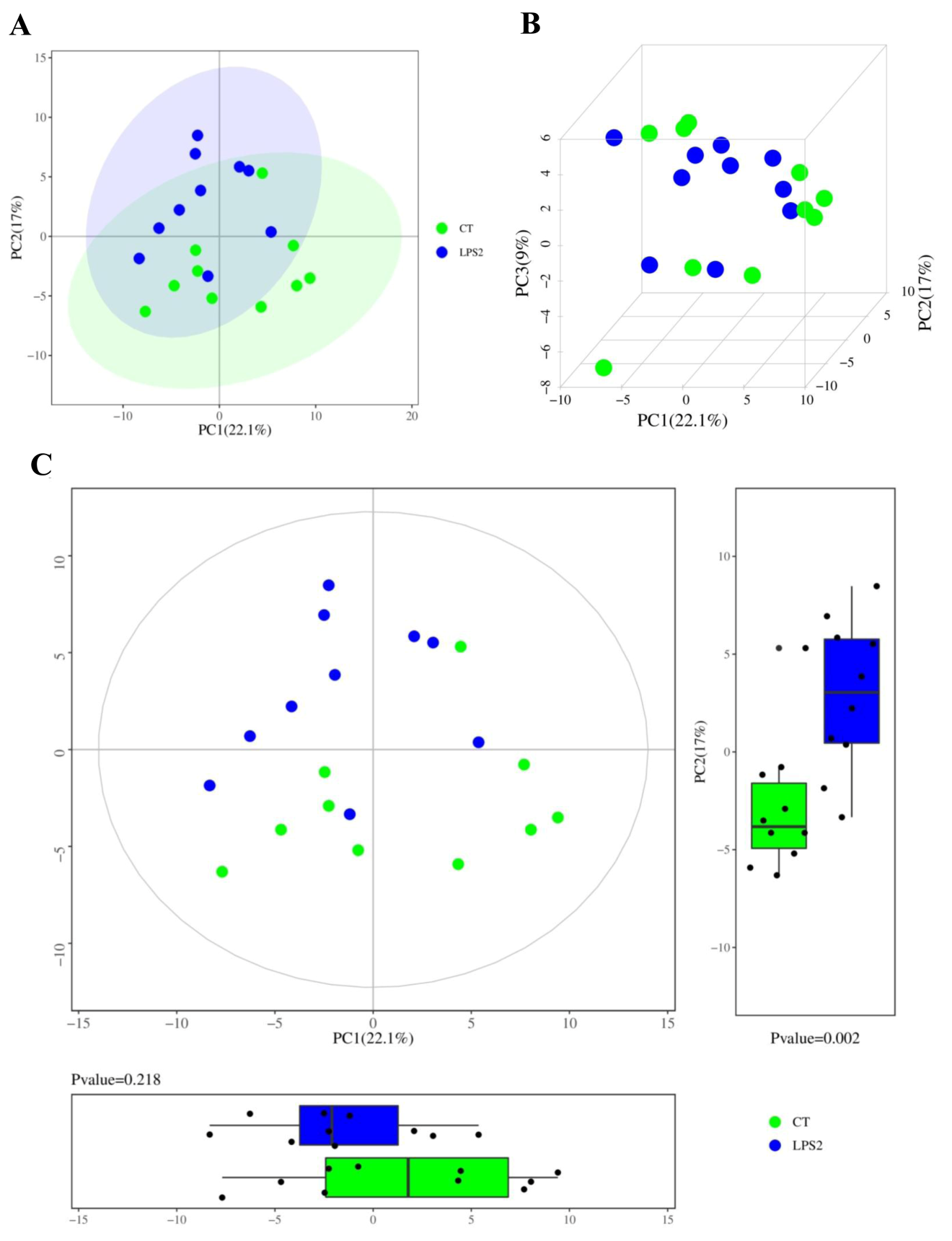


**Supplementary Figure 2 PCA score plots for the CT and LPS2 groups.** The 2D and 3D PCA scores are shown in Figure 2A and 2B, respectively. Figure 2C shows the 2D PCA score plot for the analyzed samples and box plots corresponding to the principal component scores.


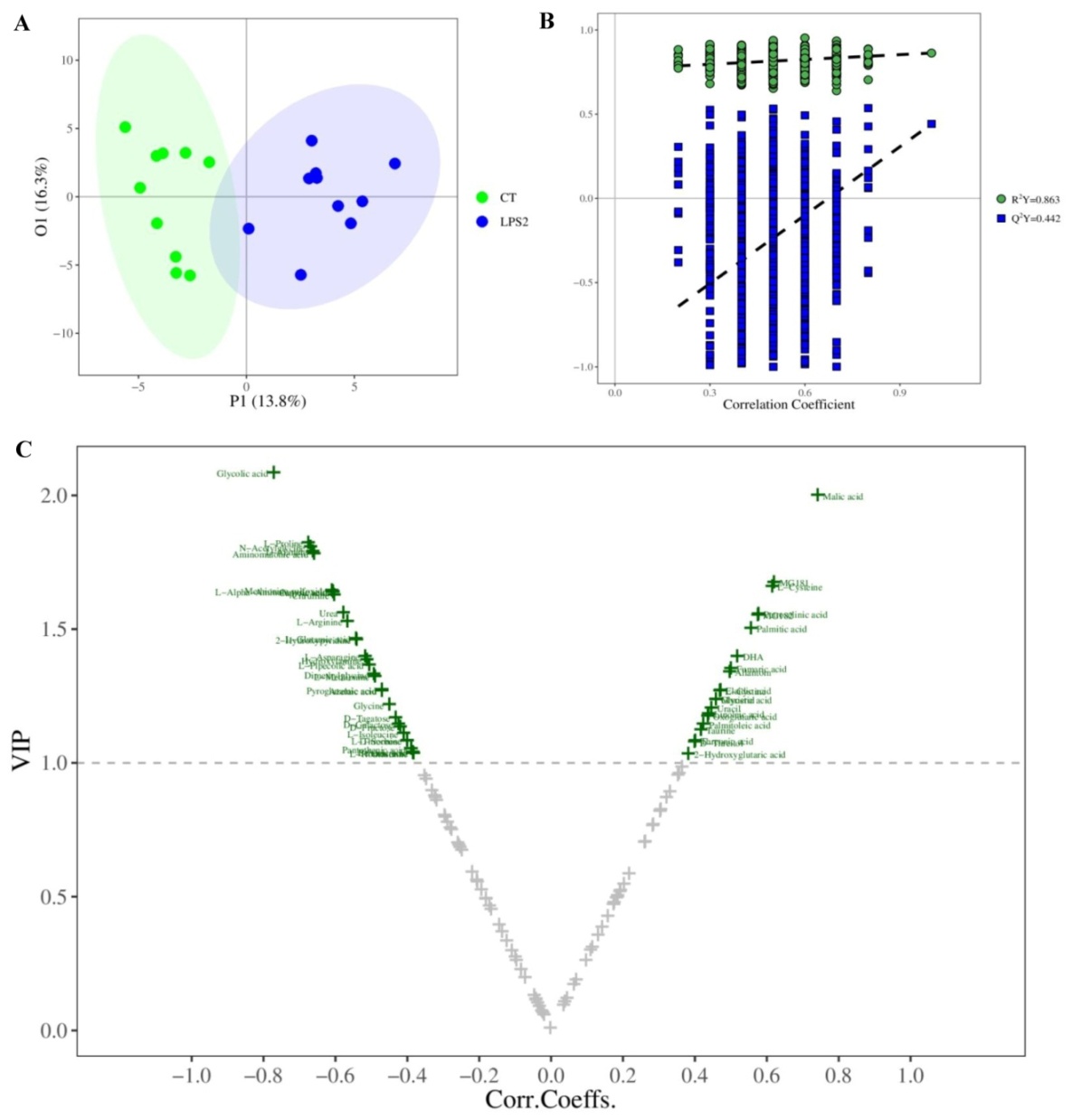


**Supplementary Figure 3 OPLS-DA score plot, permutation test results and** **volcano plot:** The OPLS-DA 2D score plot is shown in Supplementary Figure **3A**; the permutation test results are shown in Supplementary Figure **3B**. A visualization of differential metabolite profiles on a volcano plot is shown in Supplementary Figure **3C**.


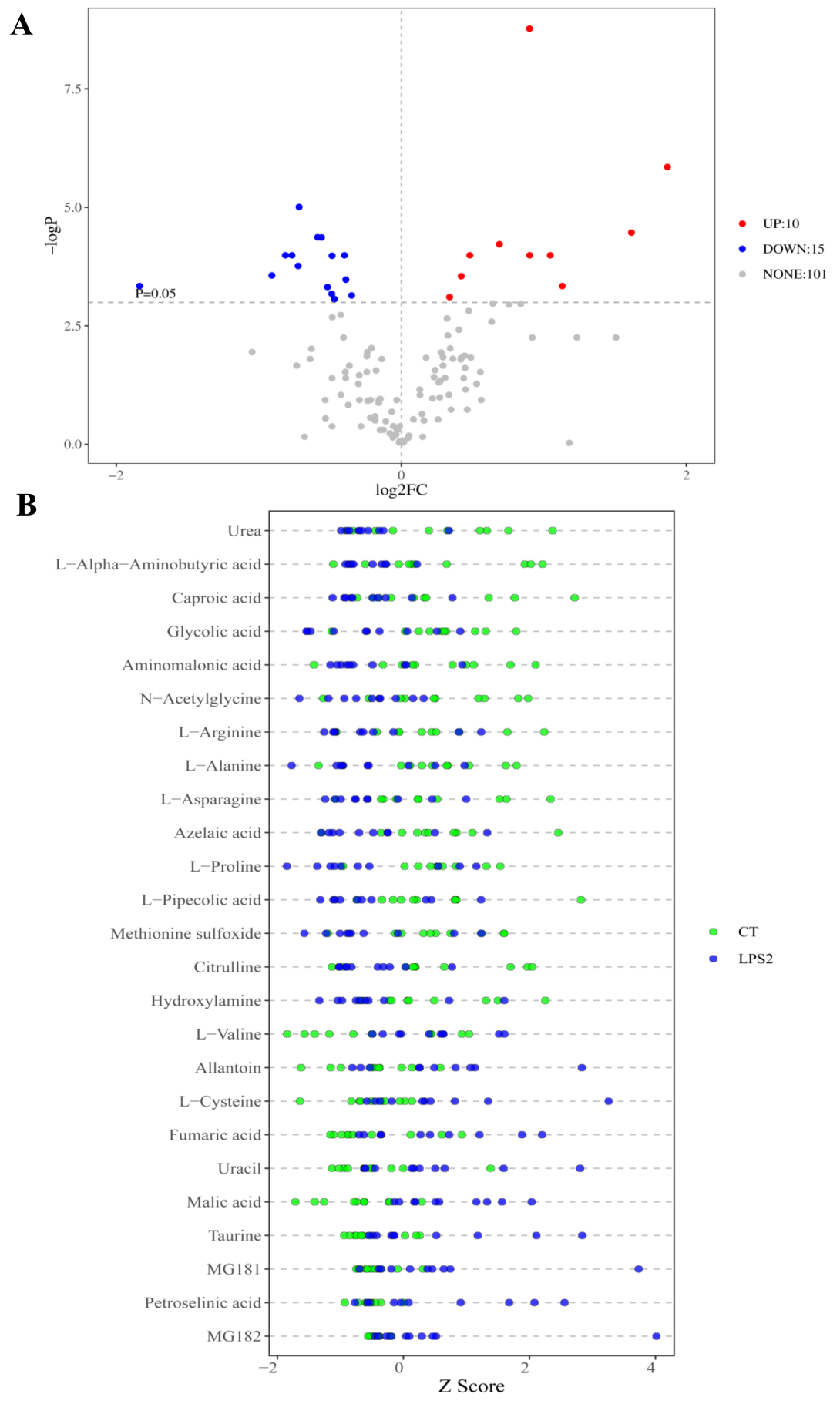


**Supplementary Figure 4A: Enhanced volcano plot and Z-score plot showing the differential metabolites selected by univariate analysis.** The volcano plot shows the screened differential metabolites based on univariate statistical analysis (Supplementary Figure 4A). In this analysis, the threshold settings for the volcano plot were as follows: (1) P < 0.05 and (2) an absolute value of log2fc > 0 (where fc is the fold change, i.e. the factor of the intergroup change). The highlighted metabolites in the upper right corner were increased and the highlighted metabolites in the upper left corner decreased in the LPS2 group compared with the CT group (**Supplementary Figure 4A**). According to the screening criteria, 25 differential metabolites were obtained by univariate statistical analysis (P < 0.05). **Supplementary Figure 4B** shows the Z-score plot of these 25 differential metabolites.


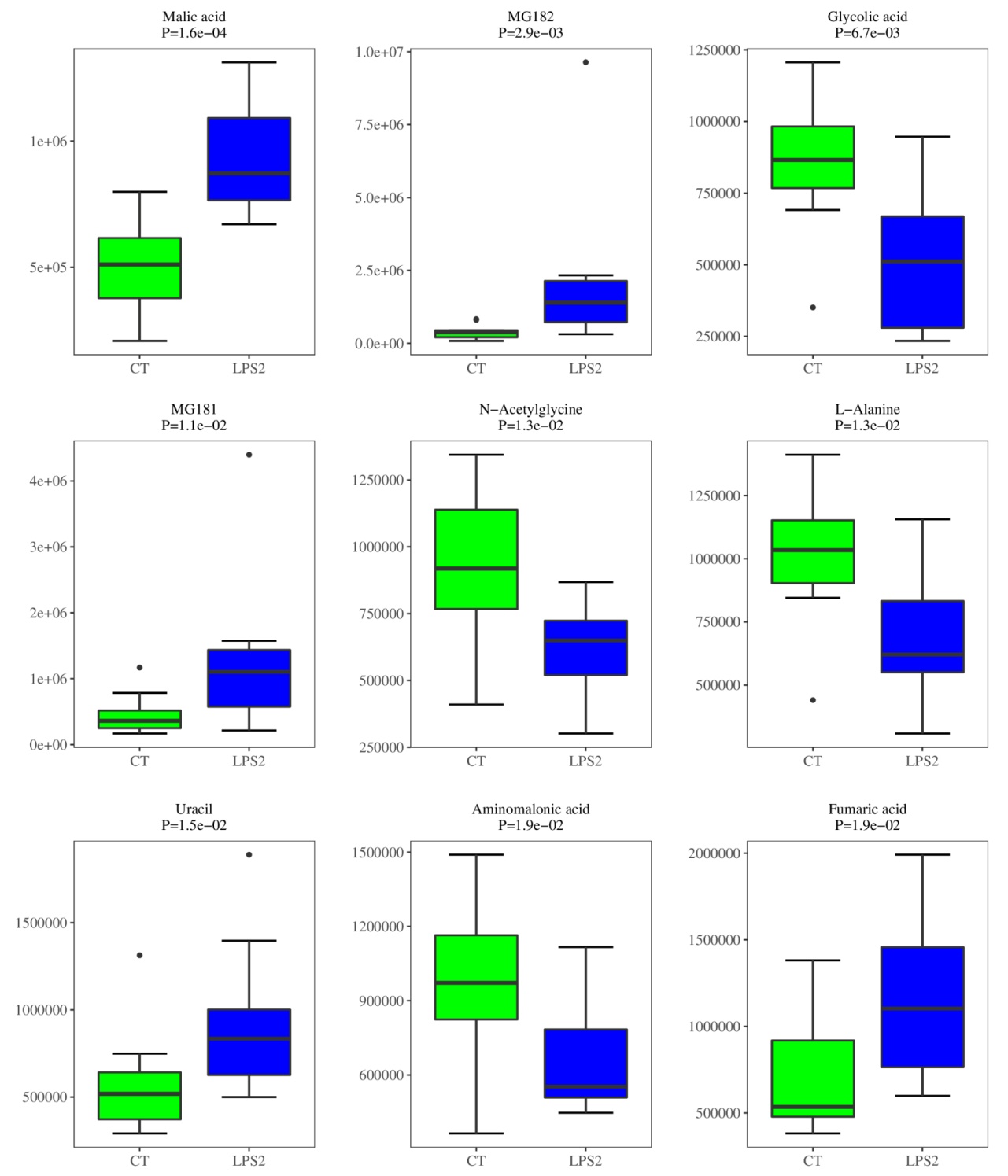


**Supplementary Figure 5 Top-ranked differential metabolites between the two groups.** The nine representative differential metabolites (top-ranked) obtained by univariate statistical analysis and their P-value rankings are shown in this figure.


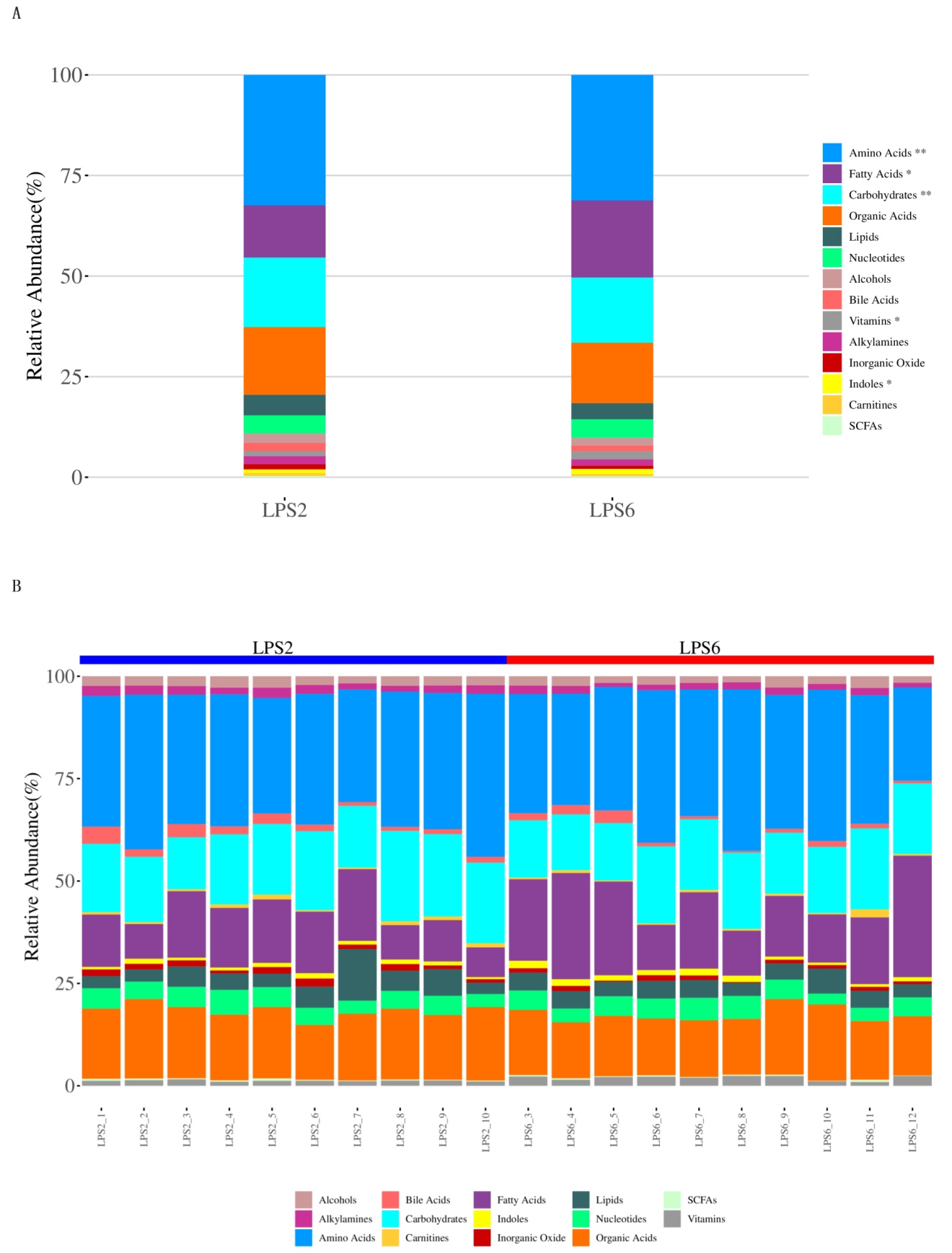


**Supplementary Figure 6 Classification of metabolites in the LPS2 group and LPS6 group.** The stacked histogram showing the relative abundance statistics of the median value of various metabolites in each group of samples is shown in **Supplementary Figure 6A**. The stacked histogram showing the relative abundances of various metabolite types in each sample is shown in **Supplementary Figure 6B**.


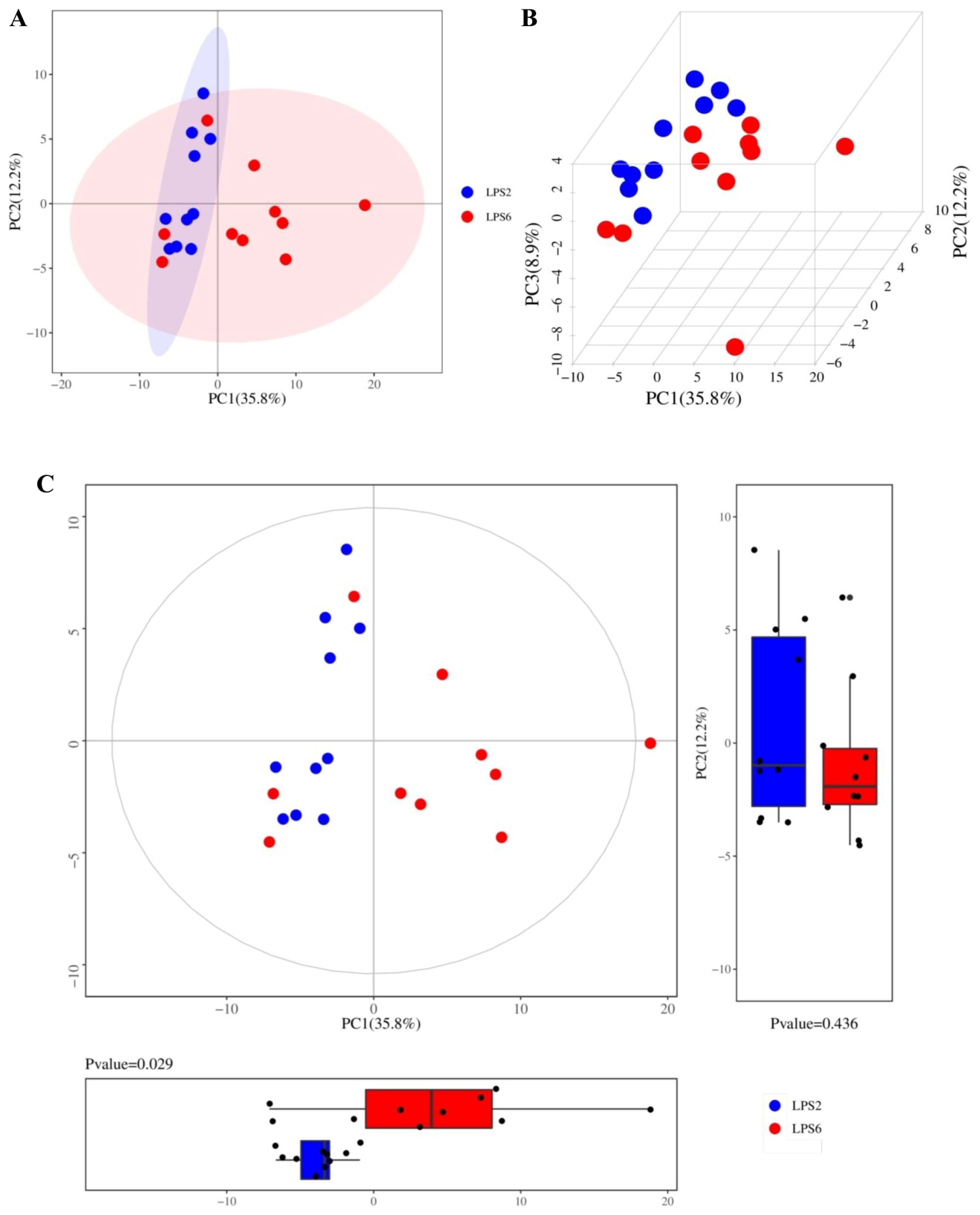


**Supplementary Figure 7 PCA score plots for the LPS2 and LPS6 groups:** The 2D and 3D PCA scores are shown in Supplementary Figure 7A and 7B, respectively. Supplementary Figure 7C shows the 2D PCA score plot of the analyzed samples and box plots corresponding to the principal component scores.


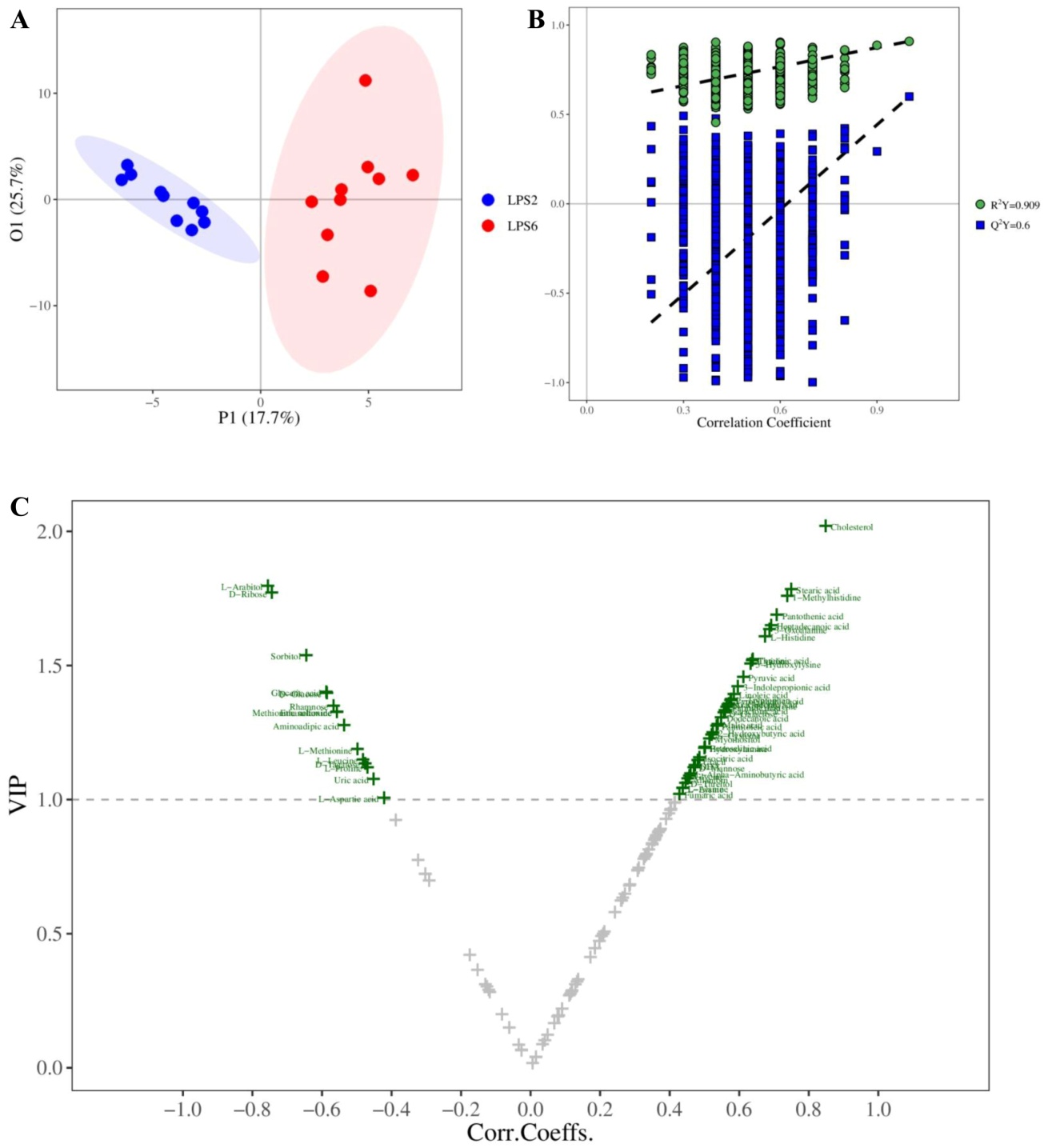


**Supplementary Figure 8 OPLS-DA score plot, permutation test results and volcano plot:** The OPLS-DA 2D score plot is shown in Supplementary Figure 8A; the permutation test results are shown in Supplementary Figure 8B; visualization of differential metabolite profiles on a volcano plot is shown in Supplementary Figure 8C.


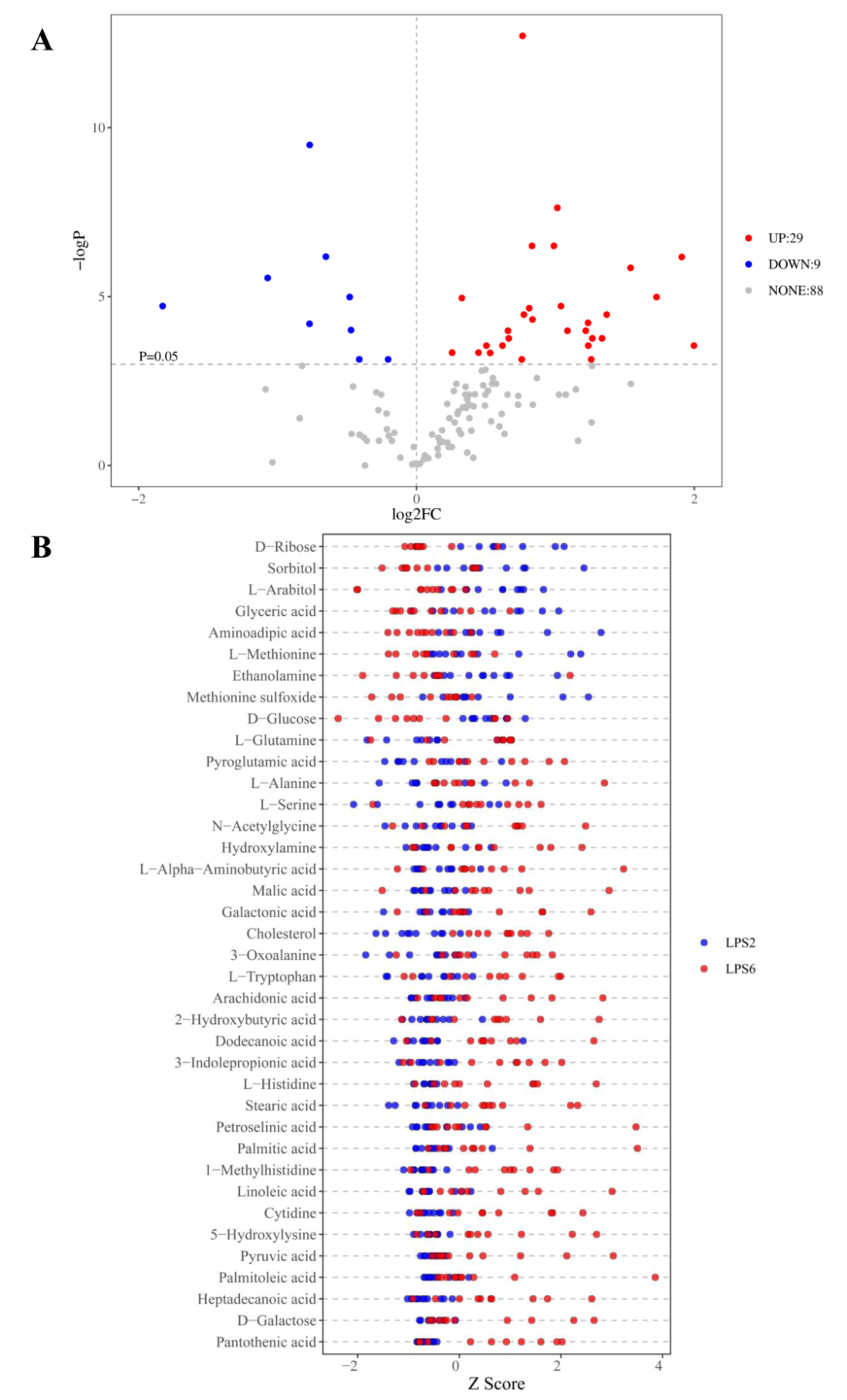


**Supplementary Figure 9 Enhanced volcano plot and Z-score plot showing the differential metabolites selected by multivariate analysis.** The volcano plot shows the screened differential metabolites based on univariate statistical analysis (Supplementary Figure 9A). In this analysis, the threshold settings for the volcano plot were as follows: (1) P < 0.05 and (2) an absolute value of log2fc > 0 (where fc is the fold change, i.e. the factor of the intergroup change). The highlighted metabolites in the upper right corner were increased and the highlighted metabolites in the upper left corner were decreased in the LPS6 group compared with the LPS2 group (Supplementary Figure 9A). According to the screening criteria, 38 differential metabolites were obtained by univariate statistical analysis (P < 0.05). Supplementary Figure 9B shows the Z-score plot of these 38 differential metabolites.


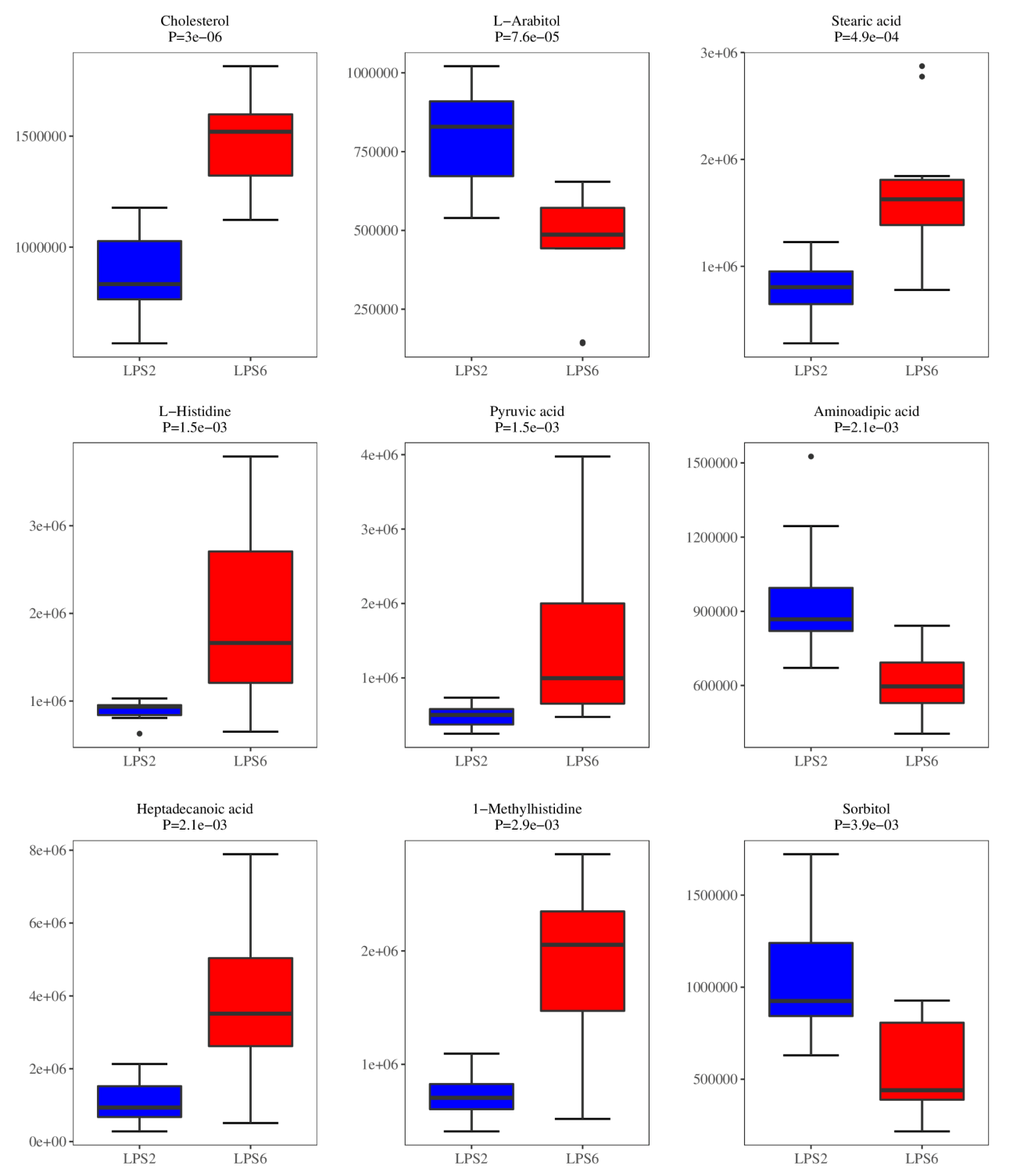


**Supplementary Figure 10 Top-ranked differential metabolites between the two groups.** The nine representative differential metabolites (top-ranked) obtained by univariate statistical analysis and their P-value rankings are shown in this figure.


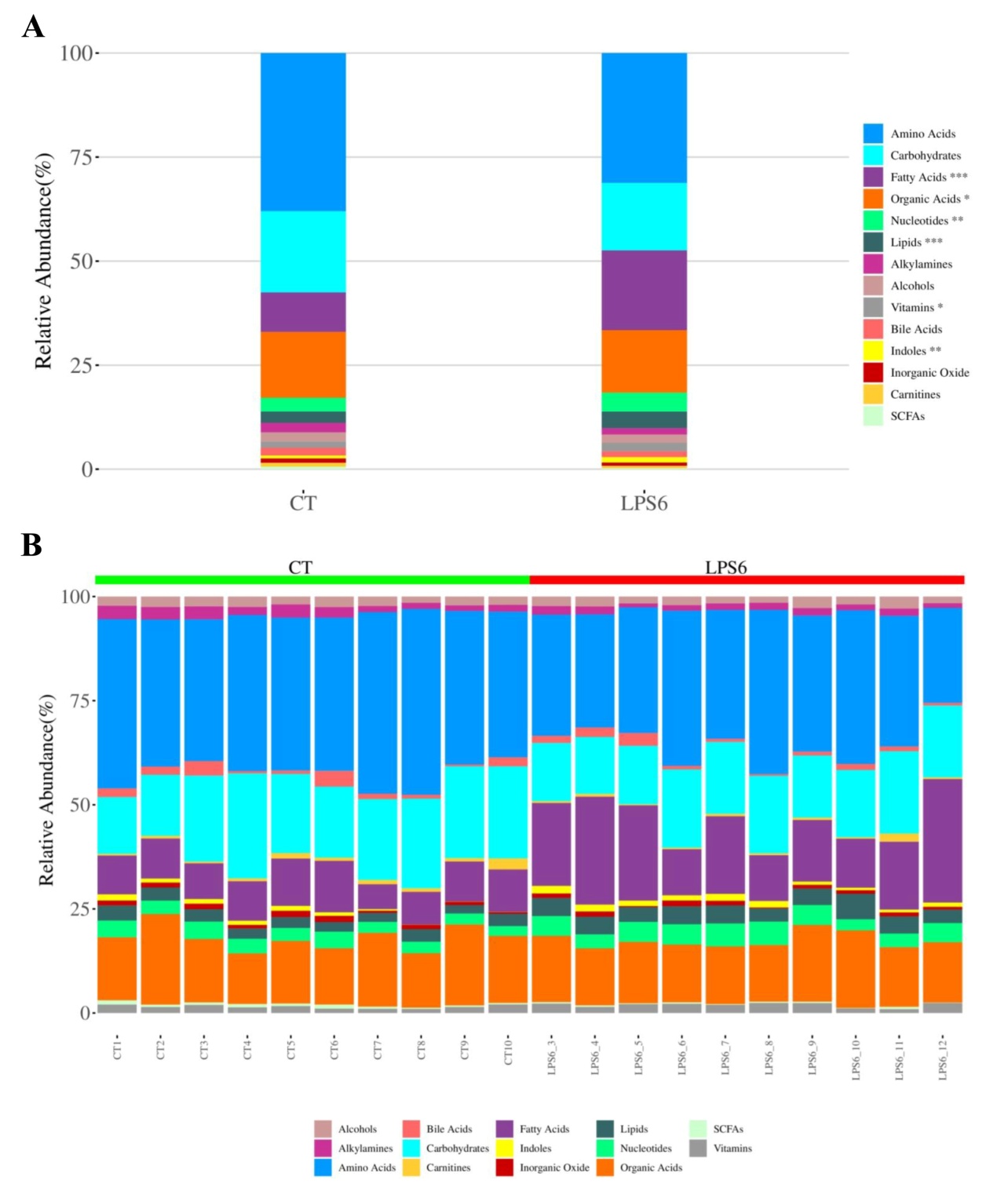


**Supplementary Figure 11 Classification of metabolites in the CT group and LPS2 group.** The stacked histogram showing the relative abundance statistics of the median value of various metabolites in each group of samples is shown in **Supplementary Figure 11A**. The stacked histogram showing the relative abundances of various metabolite types in each sample is shown in **Supplementary Figure 11B**.


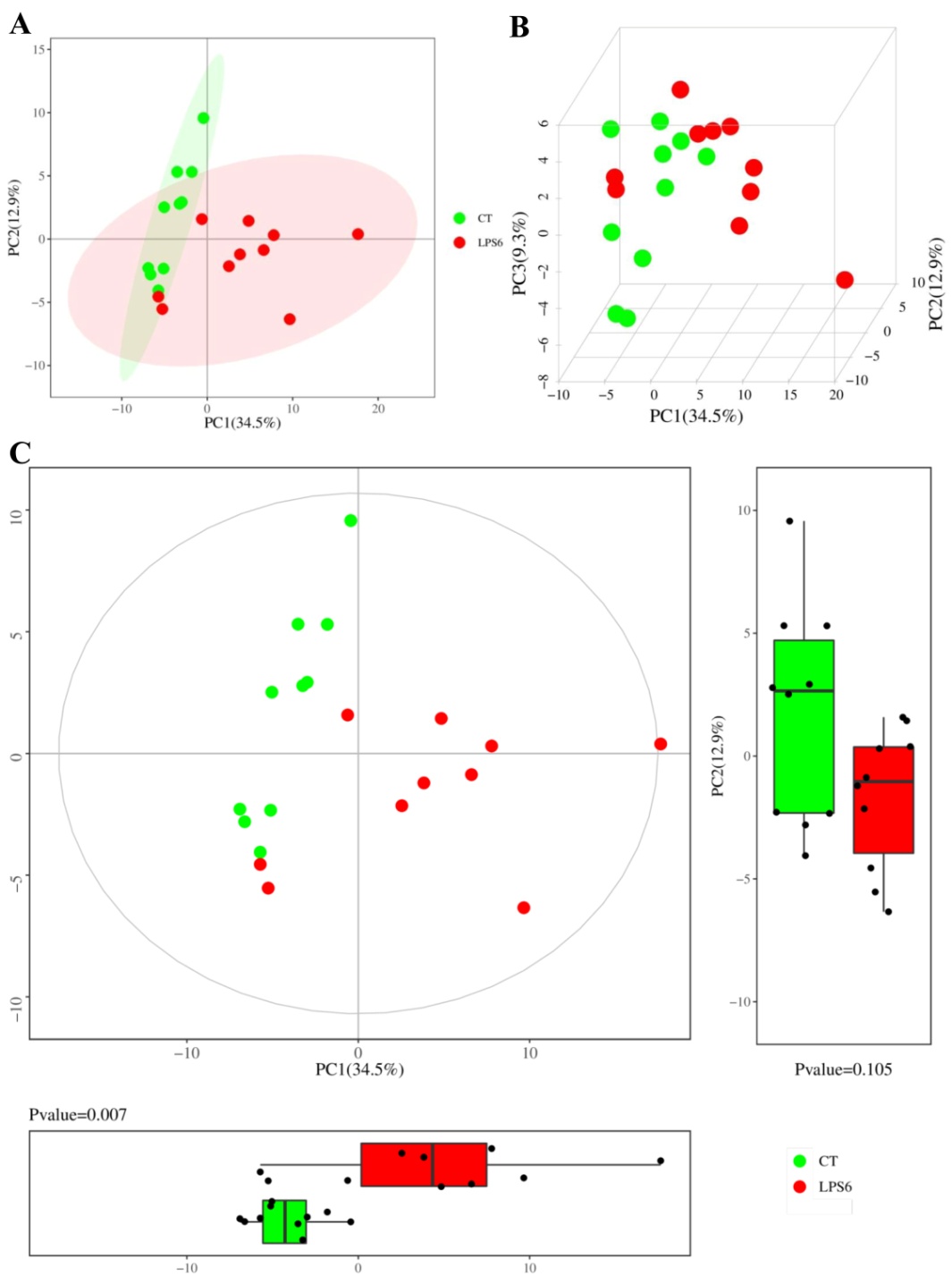


**Supplementary Figure 12 PCA score plots for the CT and LPS6 groups.** The 2D and 3D PCA scores are shown in Supplementary Figure 12A and 12B, respectively. Supplementary Figure 12C shows the 2D PCA score plot of the analyzed samples and box plots corresponding to the principal component scores.


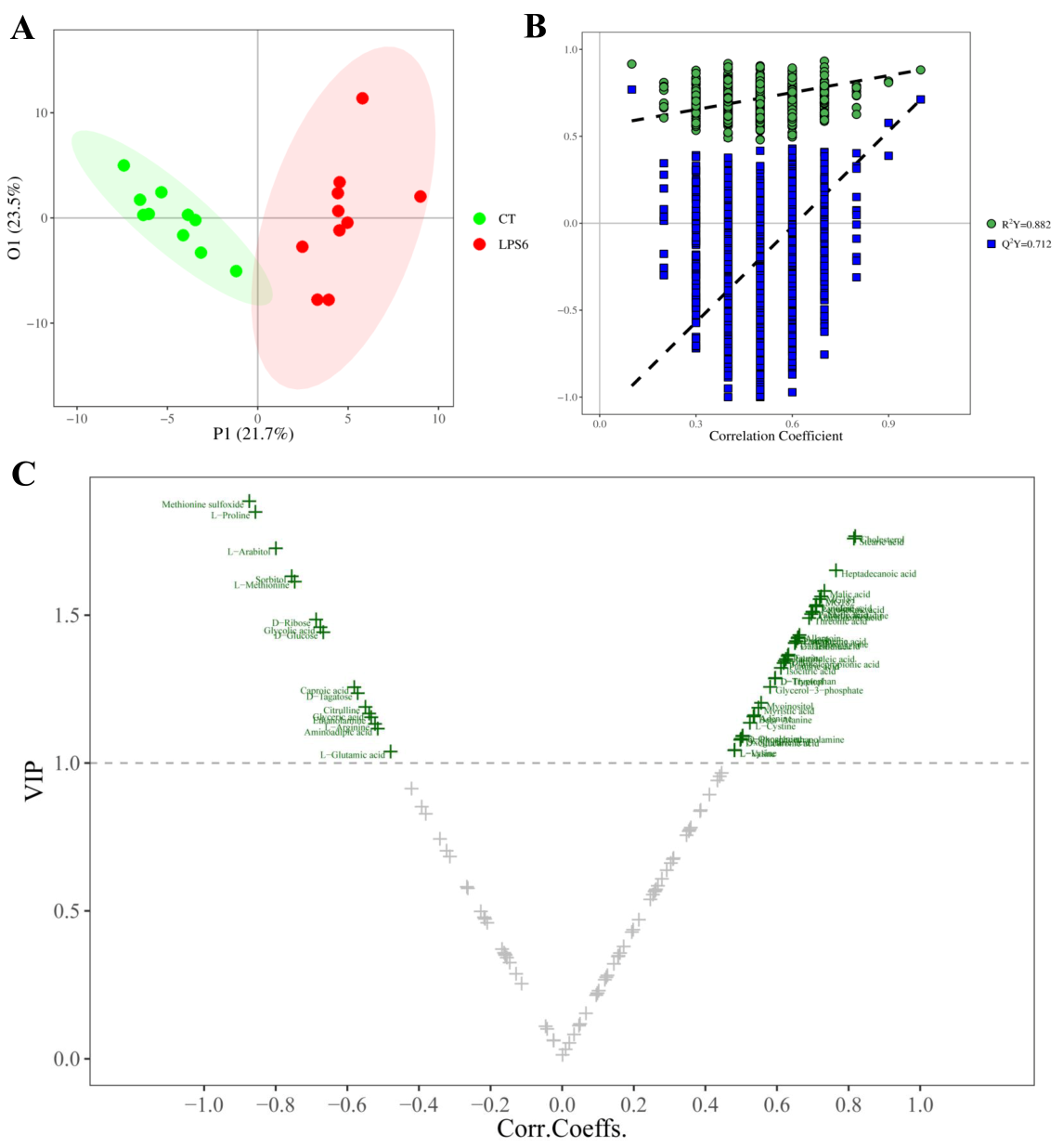


**Supplementary Figure 13 OPLS-DA score plot, permutation test results and volcano plot.** Metabolic profiles of the individuals from the two predefined groups were further differentiated using a more sophisticated multivariate statistical model, the OPLS-DA model (Supplementary Figure 13A). Permutation testing (see the Materials and Methods section for details) was employed to assess the validation of the classification model (Supplementary Figure 13B). A volcano plot (Supplementary Figure 13C) was used to screen for reliable metabolic markers.


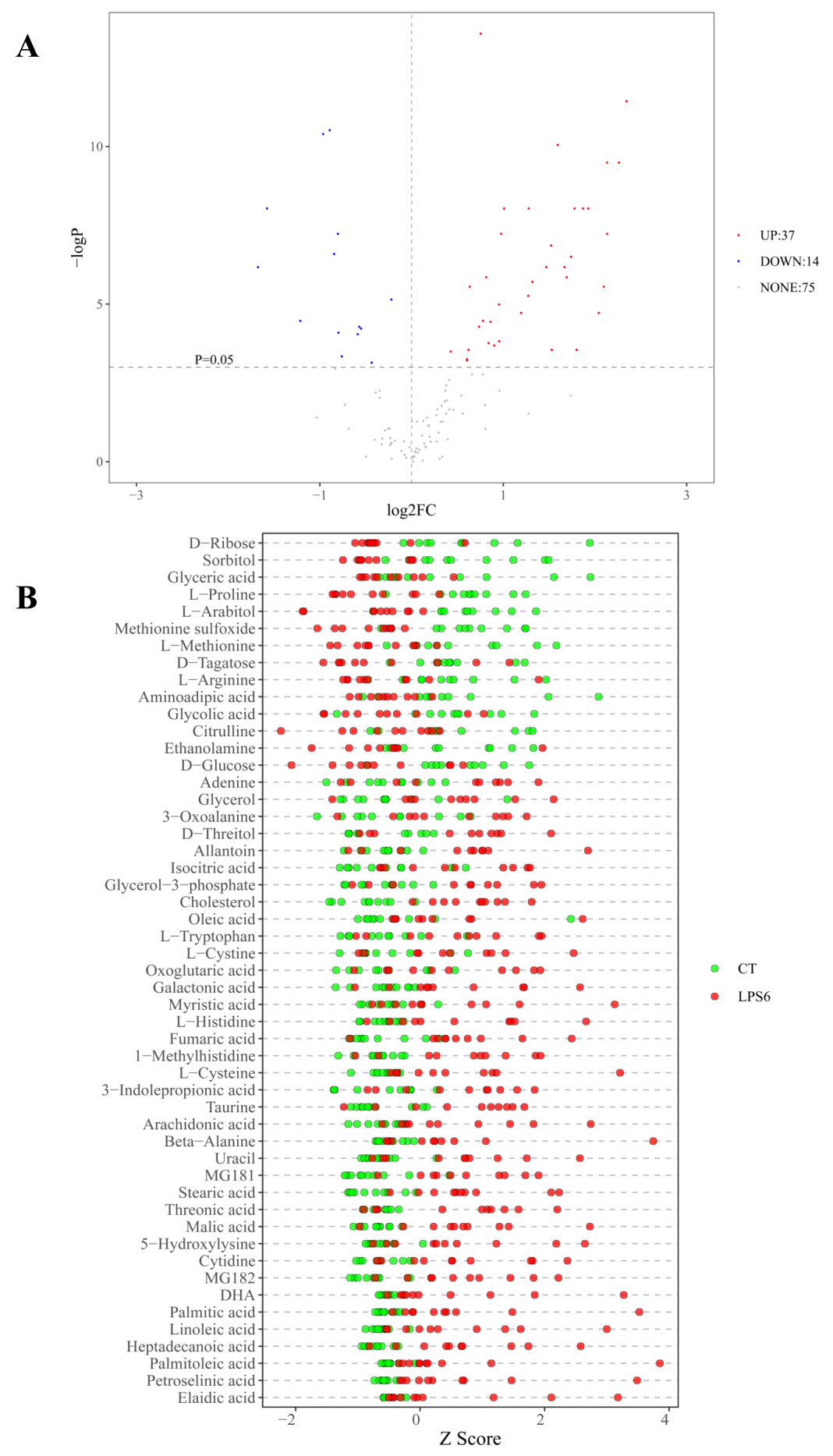


**Supplementary Figure 14 A: Enhanced volcano plot showing the differential metabolites selected by univariate analysis; B: Z-score plot of these 51 differential metabolites.** The volcano plot shows the screened differential metabolites based on univariate statistical analysis (Supplementary Figure 14A). In this analysis, the threshold settings for the volcano plot were as follows: (1) P < 0.05 and (2) an absolute value of log2fc > 0 (where fc is the fold change, i.e. the factor of the intergroup change). The highlighted metabolites in the upper right corner were increased and the highlighted metabolites in the upper left corner were decreased in the LPS6 group compared with CT group (Supplementary Figure 14A). According to the screening criteria, 51 differential metabolites were obtained by univariate statistical analysis. Supplementary Figure 14B shows the Z-score plot of these 51 differential metabolites.


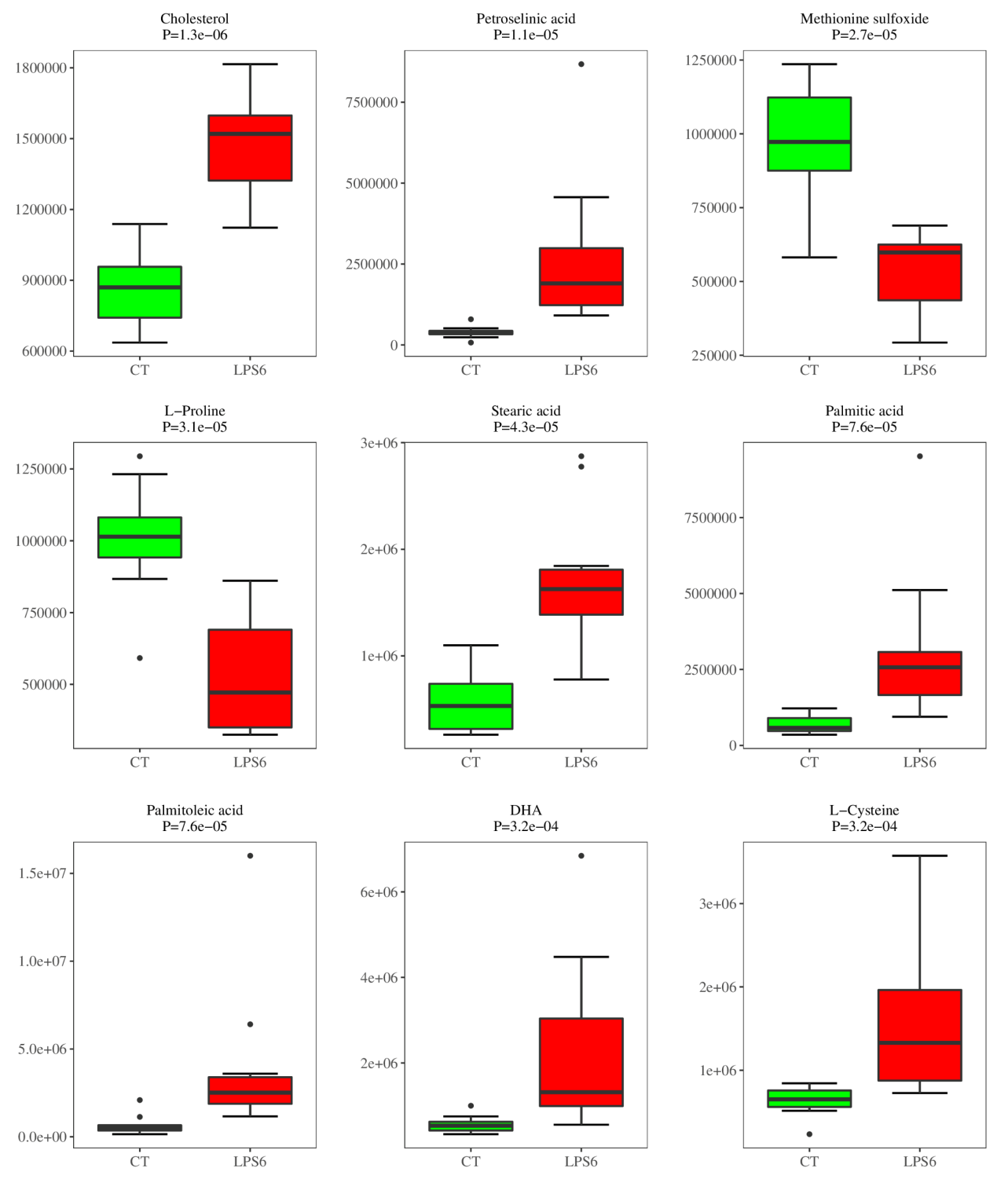


**Supplementary Figure 15 Top-ranked differential metabolites between the two groups.** The nine representative differential metabolites (top-ranked) obtained by univariate statistical analysis and their P-value rankings are shown in this figure.

**
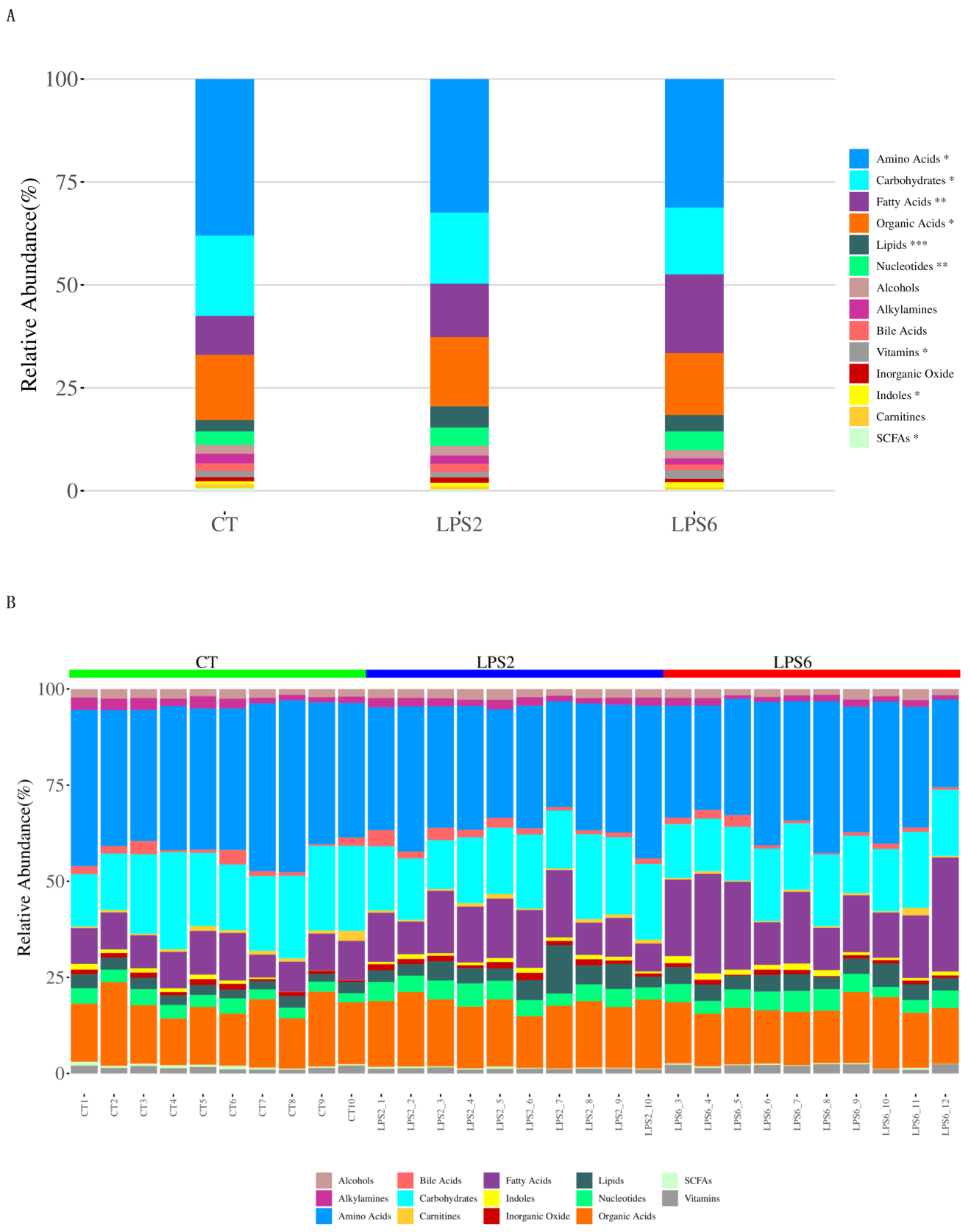
**

**Supplementary Figure 16 Classification of metabolites in the CT group, LPS2 group and LPS6 group.** The stacked histogram showing the relative abundance statistics of the median value of various metabolites in each group of samples is shown in Supplementary Figure 16A. The stacked histogram showing the relative abundances of various metabolite types in each sample is shown in Supplementary Figure 16B.


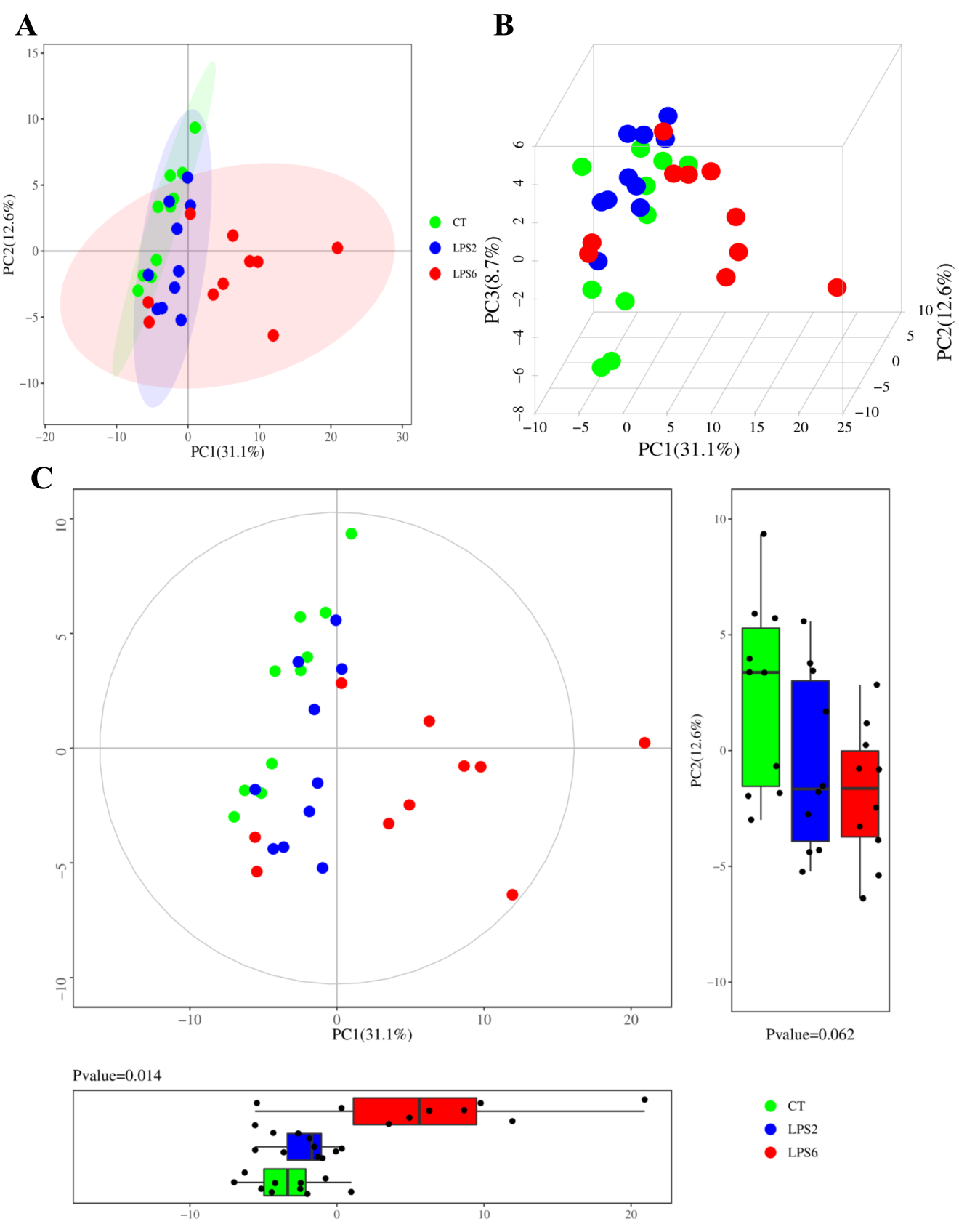


**Supplementary Figure 17 PCA score plots for the CT and LPS6 groups**. The 2D and 3D PCA scores are shown in Supplementary Figure 17A and 17B, respectively. Supplementary Figure 17C shows the 2D PCA score plot of the analyzed samples and box plots corresponding to the principal component scores.


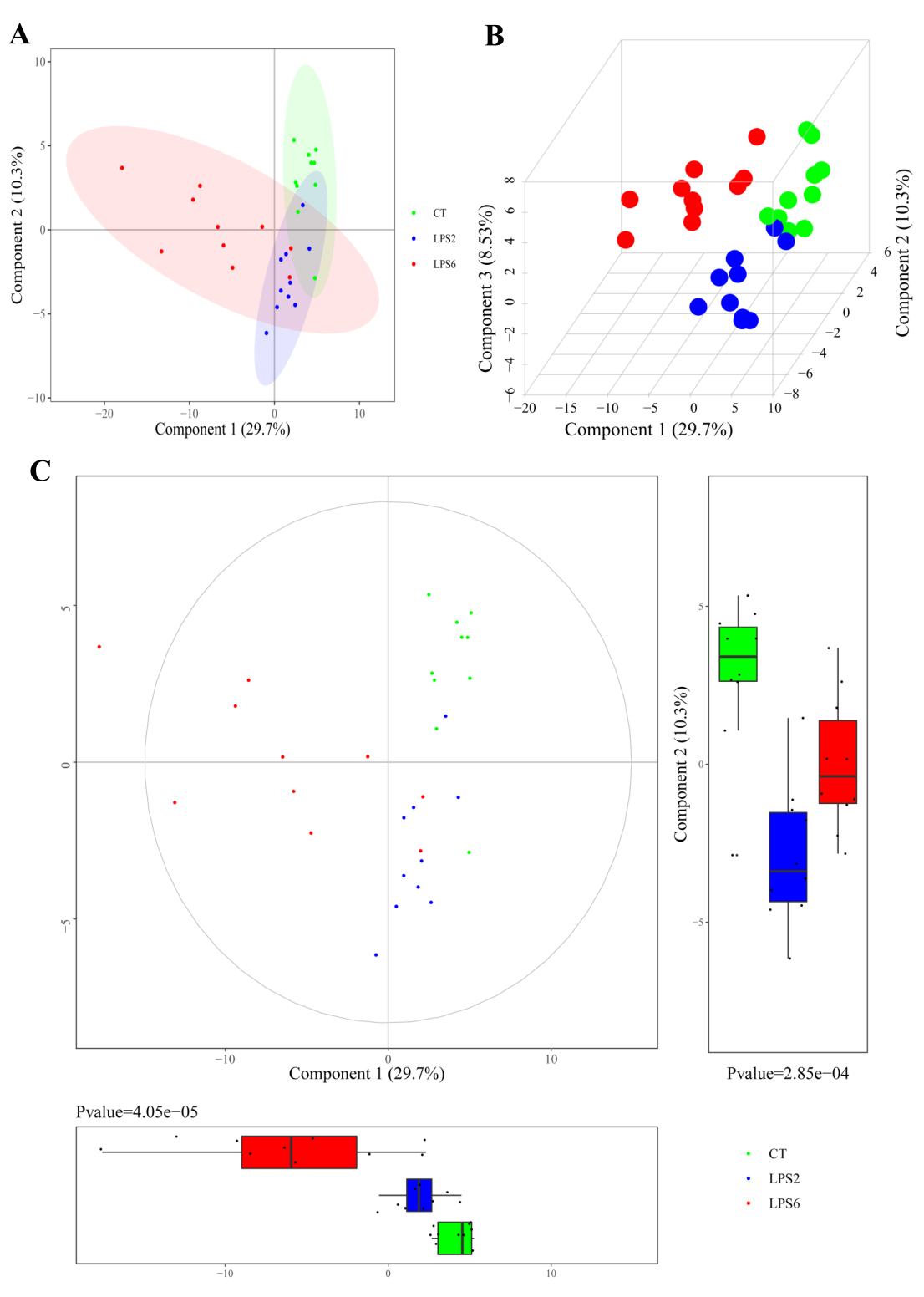


**Supplementary Figure 18 PLS-DA score plot revealing subject classifications. A: 2D PLS-DA score plot; B: 3D PLS-DA score plot; C: PLS-DA score plot with corresponding box plots.** A widely used supervised multivariate classification model, i.e., PLS-DA, was employed to perform multiple group comparisons because this model maximizes the intergroup differences (Supplementary Figure 18A, 2D PLS-DA score plot; Supplementary Figure 18B, 3D PLS-DA score plot). Supplementary Figure 18C shows the PLS-DA score plot with corresponding box plots.


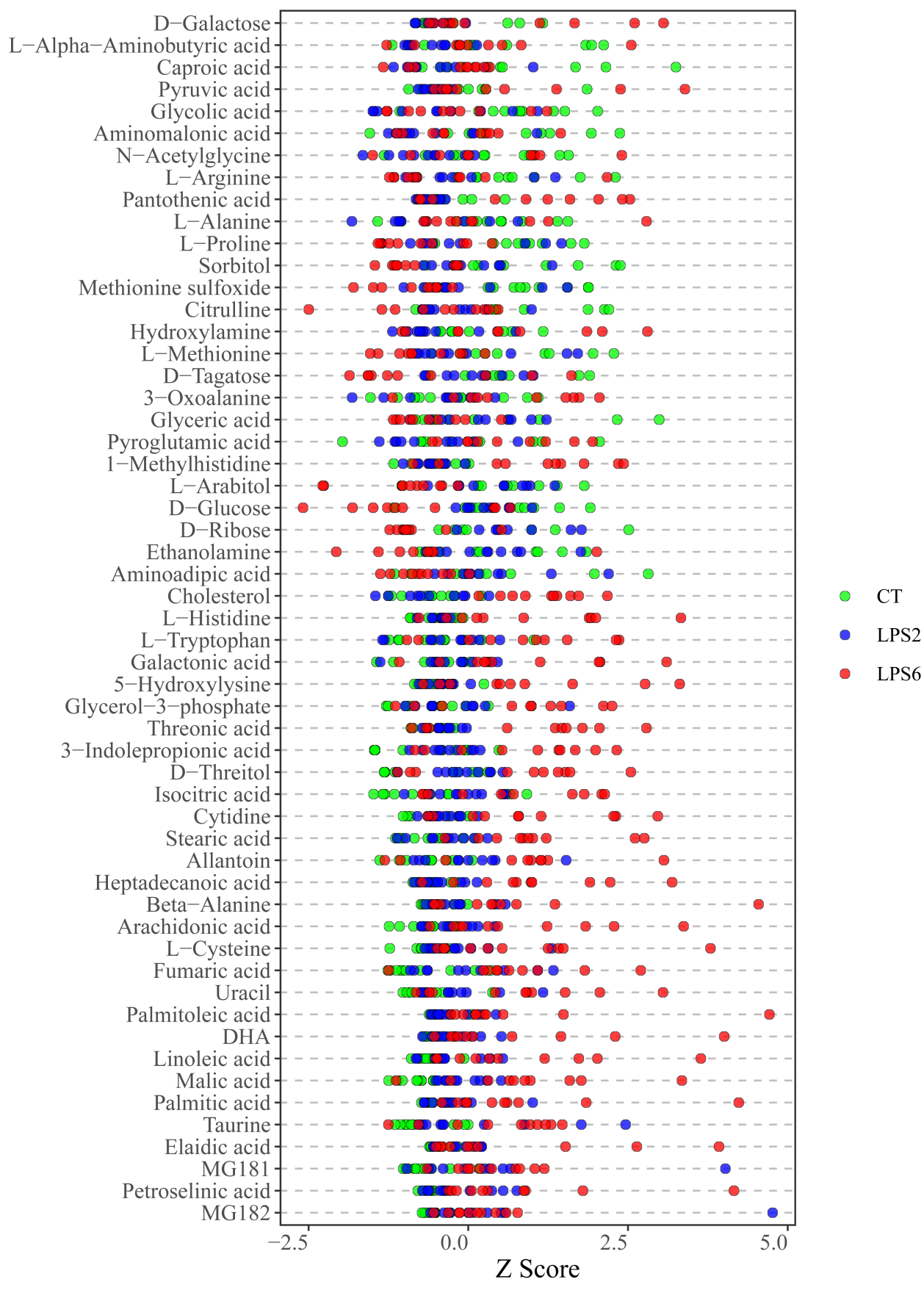


**Supplementary Figure 19 Z-score plot of the 55 differential metabolites.** 55 differential metabolites were obtained by univariate statistical analysis. Supplementary Figure 19 shows the Z-score plot of these 55 differential metabolites.

**
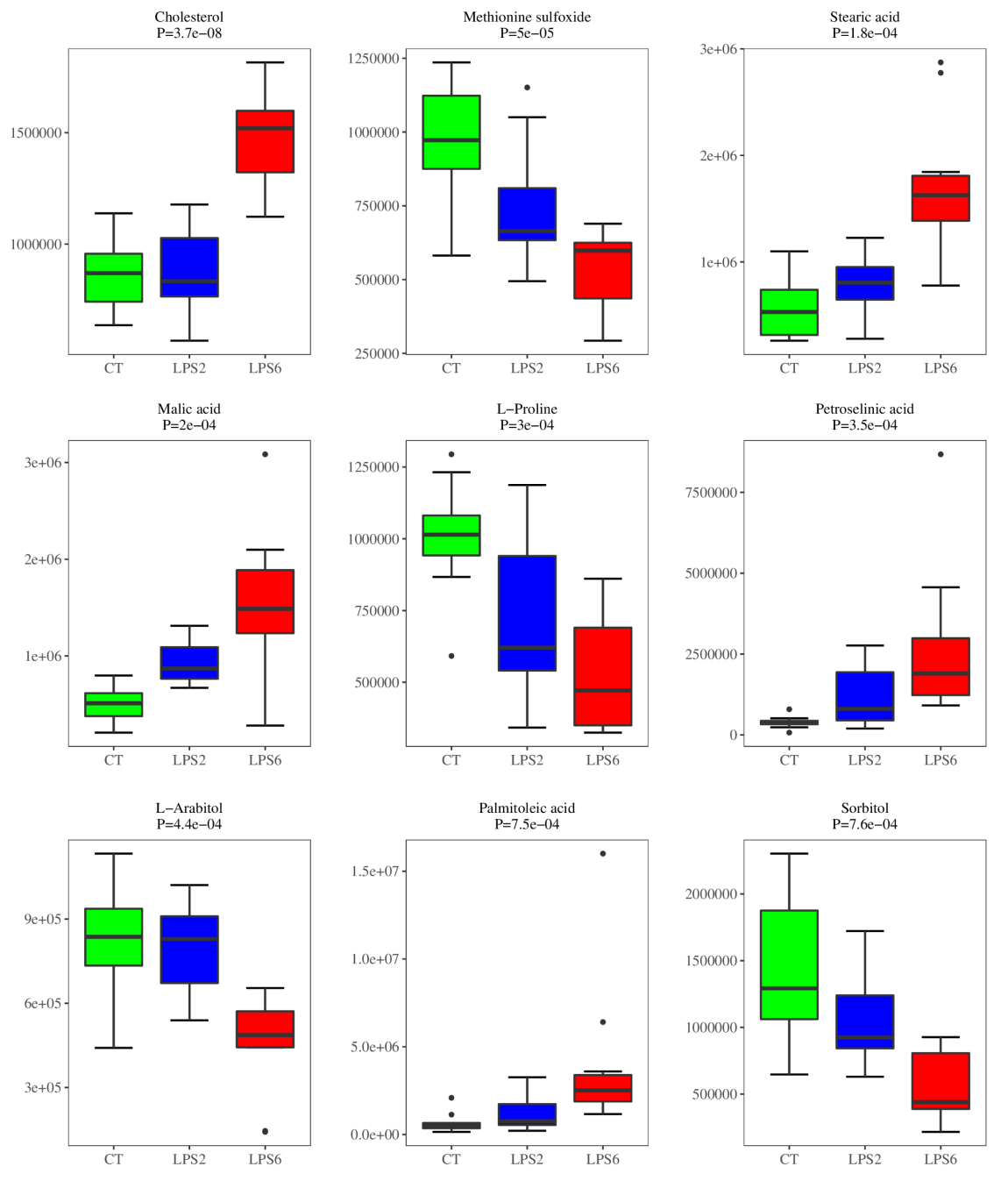
**

**Supplementary Figure 20 Top-ranked differential metabolites among the three groups.** The nine representative differential metabolites (top-ranked) obtained from univariate statistical analysis and their P-value rankings are shown in this figure.
